# Hypertriglyceridemia: Causes, Consequences, Diagnosis, and Management

**DOI:** 10.1002/mco2.70956

**Published:** 2026-09-30

**Authors:** Shanshan Qi, Weiwei Chen, Kunhao Chen, Song Yang, Sibo Wang, Tongtian Ni, Dan Xu, Enqiang Mao, Erzhen Chen, Ying Chen

**Affiliations:** ^1^ Department of Emergency Ruijin Hospital Shanghai Jiao Tong University School of Medicine Shanghai China; ^2^ Emergency & Critical Care Medicine Center Ruijin Hospital Shanghai Jiao Tong University School of Medicine Shanghai China; ^3^ Shanghai Institute of Aviation Medicine Shanghai Jiao Tong University Medical School Affiliated Ruijin Hospital Shanghai China

**Keywords:** acute pancreatitis, atherosclerotic cardiovascular disease, chylomicronemia, hypertriglyceridemia, triglyceride‐rich lipoproteins

## Abstract

Managing hypertriglyceridemia (HTG) requires more than lowering triglyceride (TG) levels. Two related but clinically distinct risk domains often guide management: residual atherosclerotic cardiovascular disease (ASCVD) risk and acute pancreatitis (AP) risk. Their relative importance varies among patients and may change over time. Residual ASCVD risk is driven in part by the burden of atherogenic apolipoprotein B (apoB)‐containing particles, particularly TG‐rich lipoproteins and their cholesterol‐enriched remnants, whereas AP risk increases with severe HTG and rises further in extreme HTG, in which chylomicron accumulation, free fatty acid‐mediated lipotoxicity, and inflammation contribute to pancreatic injury. Because similar TG values can arise from different combinations of genetic susceptibility, metabolic dysfunction, and other secondary factors, TG alone may not fully characterize clinical risk or provide a sufficient basis for treatment selection. In this review, we examine the genetic and metabolic determinants of HTG and consider how TG, apoB, remnant cholesterol, chylomicronemia, and secondary causes can be integrated into clinical assessment. We also evaluate established treatments and emerging therapies targeting apolipoprotein C‐III, angiopoietin‐like protein 3, and related pathways. This risk‐based framework can help clinicians tailor treatment to each patient's ASCVD and pancreatitis risk profile.

## Graphical abstract

Hypertriglyceridemia (HTG) arises from the interplay between genetic susceptibility and secondary or precipitating factors, leading to dysregulated triglyceride‐rich lipoprotein (TRL) metabolism. Increased TRL production and impaired clearance promote distinct risk phenotypes: accumulation of apolipoprotein B(apoB)‐containing TRL remnants contributes to residual atherosclerotic cardiovascular disease (ASCVD) risk, whereas extreme HTG with chylomicronemia predisposes to HTG‐associated acute pancreatitis (HTG‐AP). Emerging therapies targeting key regulators of TRL metabolism, including apolipoprotein C‐III (apoC‐III) and angiopoietin‐like protein 3 (ANGPTL3), may provide new opportunities for phenotype‐based risk reduction and precision management.

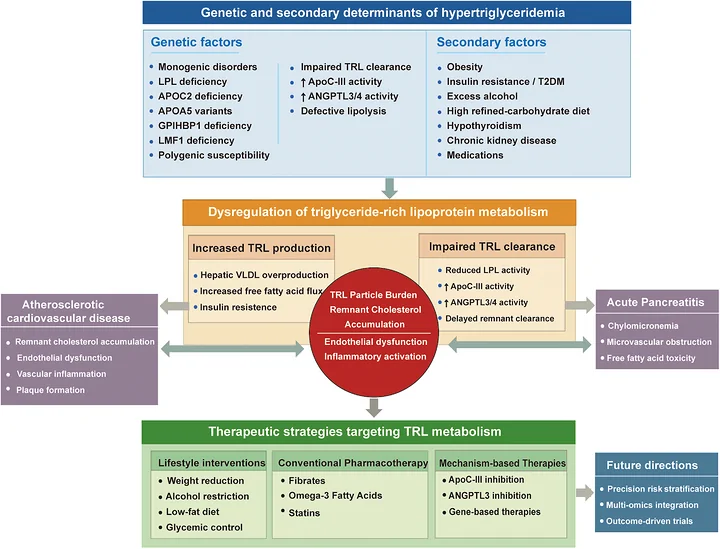

## 1 Introduction

Hypertriglyceridemia (HTG) is a common lipid disorder characterized by elevated circulating triglyceride (TG) levels, with clinical consequences that vary according to disease severity, lipoprotein phenotype, and underlying metabolic context [1]. With the increasing prevalence of obesity, type 2 diabetes, insulin resistance, and related metabolic disorders, the global burden of HTG has risen over recent decades [2, 3, 4]. The condition encompasses two major, potentially overlapping risk domains with distinct pathophysiological mechanisms and management priorities. Given the differences in TG thresholds and terminology across current guidelines and consensus statements, this review uses severe HTG to refer to fasting TG levels of 5.6–11.3 mmol/L (500–999 mg/dL) and chylomicronemia‐range HTG, also termed extreme HTG in recent consensus statements, to refer to fasting TG levels ≥11.3 mmol/L (≥1000 mg/dL), unless otherwise specified. The risk of HTG‐associated acute pancreatitis (HTG‐AP) increases in severe HTG and becomes particularly pronounced in extreme HTG, in which free fatty acid (FFA)‐mediated lipotoxicity and inflammatory pathways contribute to pancreatic injury [5, 6]. In contrast, persistent elevations in TG‐rich lipoproteins (TRLs) and their remnants are associated with residual atherosclerotic cardiovascular disease (ASCVD) risk through the accumulation of remnant cholesterol (RC) and atherogenic apolipoprotein B (apoB)‐containing particles [7, 8]. HTG is also commonly observed in the context of broader metabolic dysfunction and frequently coexists with chronic metabolic diseases such as metabolic dysfunction‐associated steatotic liver disease (MASLD), suggesting that these conditions may share common metabolic drivers [9].

Most cases of HTG are not explained by a single‐gene defect. Instead, genetic susceptibility often interacts with secondary factors such as obesity, insulin resistance, alcohol consumption, certain medications, and pregnancy [10, 11, 12]. This interplay helps explain why patients differ in lipoprotein profiles, TRL clearance, and susceptibility to organ‐specific complications. Impaired clearance and overproduction of TRLs may lead to accumulation of chylomicrons (CMs) or remnants and disrupt fatty acid metabolism [13]. Excess exposure to FFAs can promote lipotoxicity, mitochondrial dysfunction, oxidative stress, and endoplasmic reticulum (ER) stress, thereby activating inflammatory signaling pathways [14, 15, 16, 17]. From the perspective of lipoprotein metabolism, HTG is not merely an isolated elevation in TG levels but reflects an increased burden of TRLs and their remnants in the circulation [18]. In some individuals, these responses are closely linked to HTG‐AP risk; in others, the dominant concern is prolonged exposure to atherogenic remnant particles and residual ASCVD risk. Accordingly, consideration of lipoprotein composition alongside organ‐specific outcomes may help distinguish pancreatitis‐predominant from ASCVD‐predominant HTG.

HTG‐AP represents one of the most severe acute complications of HTG. Although gallstone disease and alcohol consumption remain the leading causes of acute pancreatitis (AP) overall [19], HTG is increasingly recognized as an important etiology [20], with several East Asian cohort studies suggesting an increasing clinical burden in these settings [21, 22, 23]. Patients with HTG‐AP often show more pronounced metabolic disturbances than those with pancreatitis from other causes and may have higher risks of systemic inflammatory response syndrome (SIRS), organ failure, and recurrence [24, 25]. In this setting, pancreatitis risk rises substantially at extreme TG levels, particularly in the presence of persistent chylomicronemia (PC) [5]. By contrast, the cardiovascular consequences of HTG are shaped less by short‐term extremes in TG levels than by the cumulative duration and composition of TRL exposure [26]. Thus, pancreatitis and ASCVD represent distinct but potentially overlapping clinical expressions of HTG, differing not only in target‐organ involvement but also in their temporal patterns, predominant lipoprotein drivers, and risk trajectories.

Despite major advances in genetic diagnosis, lipoprotein biology, and targeted therapeutics, HTG remains difficult to manage in clinical practice [5, 27]. Current diagnostic and risk stratification approaches still use TG concentration as a central marker of HTG severity, yet a single TG measurement cannot adequately capture etiological heterogeneity, lipoprotein composition, RC burden, chylomicronemia status, or organ‐specific risk [8, 26]. These mechanistic and clinical differences support a shift from a purely TG‐centric view of HTG toward a phenotype‐oriented framework [8, 28, 29, 30]. Within this framework, pancreatitis‐predominant and ASCVD‐predominant HTG should not be viewed as rigid diagnostic categories, but as partially overlapping clinical trajectories characterized by different dominant risk markers and therapeutic priorities. Table 1 summarizes the key clinical, biochemical, etiological, and therapeutic distinctions between these two phenotypes. This distinction matters because patients with similar TG levels may differ substantially in lipoprotein phenotype, metabolic background, genetic susceptibility, and organ‐specific outcomes, a divergence that is difficult to reconcile with a framework anchored solely to TG concentration. The therapeutic implications follow directly from this perspective: TG lowering is not always the sole objective. Depending on the clinical context, the dominant priority may be prevention of recurrent AP, reduction of atherogenic apoB‐containing particle burden, or both. The development and clinical introduction of therapies targeting apolipoprotein C‐III (apoC‐III), together with ongoing investigation of angiopoietin‐like protein 3 (ANGPTL3)‐directed approaches, have further highlighted the need for refined phenotyping and patient selection. These strategies provide approved or investigational therapeutic opportunities for selected patients with severe or extreme HTG, particularly those with familial chylomicronemia syndrome (FCS) or PC. However, the strength of evidence, regulatory status, and appropriate target populations differ among individual agents. Important clinical questions therefore remain regarding patient prioritization, the optimal timing of targeted treatment, and whether therapeutic success should be defined by TG lowering, prevention of AP, reduction in ASCVD risk, or a combination of these outcomes. Additional evidence is also needed to establish their long‐term clinical benefits and safety [31, 32, 33, 34].

**TABLE 1:** Comparison of pancreatitis‐predominant and ASCVD‐predominant HTG phenotypes.

Feature	Pancreatitis‐predominant HTG phenotype	ASCVD‐predominant HTG phenotype
Major clinical risk	AP, particularly HTG‐AP	Residual ASCVD risk
Typical TG pattern	Usually extreme HTG (TG ≥1000 mg/dL), which may be persistent, recurrent, or markedly fluctuating. However, pancreatitis risk increases progressively across the severe HTG range, and no absolute TG threshold determines whether AP will occur.	Usually persistent mild‐to‐moderate HTG. ASCVD risk is driven more by the number and cholesterol content of apoB‐containing particles than by the TG concentration itself.
Predominant lipoprotein abnormality	Persistent or intermittent chylomicronemia; in MCS, concomitant VLDL accumulation is common.	Increased VLDL, TRL remnants, and remnant cholesterol
apoB profile	In FCS, apoB is usually normal or low, with discordance between TG and apoB levels; in MCS, apoB may be elevated.	apoB is usually elevated or relatively high.
Major causes and genetic background	A minority of cases are caused by biallelic defects in the LPL pathway; most result from polygenic susceptibility superimposed on secondary factors.	Predominantly associated with polygenic susceptibility and a metabolic background characterized by insulin resistance.
Diagnostic clues	Chylomicronemia‐range TG elevation that may be persistent or markedly fluctuating, together with persistent or intermittent chylomicronemia, and a history of AP or recurrent AP, with assessment for secondary triggers and genetic clues.	Persistent TG elevation accompanied by increased apoB, non‐HDL‐C, or remnant cholesterol, particularly in the setting of diabetes, obesity, MASLD, or established ASCVD.
Management goals	Reduce markedly elevated TG levels and chylomicron burden, correct secondary factors, and prevent incident or recurrent HTG‐AP.	Reduce the burden of apoB‐containing particles and remnant cholesterol, and comprehensively lower long‐term ASCVD risk.
Treatment strategies	Low‐fat or very‐low‐fat dietary therapy according to TG severity; elimination of secondary triggers; optimization of glycemic control; fibrates and prescription omega‐3 fatty acids when appropriate; and apoC‐III–targeted therapy in selected patients according to approved indications and clinical context.	Statin‐based therapy; ezetimibe and/or PCSK9 inhibition when indicated to achieve LDL‐C, non‐HDL‐C, and apoB goals; icosapent ethyl in eligible high‐risk patients; and comprehensive control of cardiovascular risk factors.
Clinical overlap	Increased apoB‐containing particle burden and residual ASCVD risk may coexist.	AP risk may also be present when TG levels become markedly elevated, particularly with chylomicronemia.

*Note*: The two phenotypes presented in this table constitute a conceptual classification based on the predominant clinical risk and should not be interpreted as mutually exclusive formal diagnostic entities. Individual patients may have both pancreatitis risk and residual ASCVD risk, and the predominant risk phenotype may shift over time according to TG levels, the presence and persistence of chylomicronemia, metabolic status, genetic background, treatment response, and secondary factors. TG thresholds should therefore be used as supportive clinical tools rather than as substitutes for individualized assessment of chylomicronemia status, apoB‐containing particle burden, previous clinical events, and overall cardiometabolic risk. The table was developed by the authors from guidelines and expert consensus statements on HTG, studies of TRL metabolism and apoB‐containing particles, research differentiating FCS from MCS, and evidence concerning residual ASCVD risk. Key references providing the conceptual framework for this table are listed below. Detailed supporting citations for individual statements are provided in the corresponding sections of the main text [5, 8, 12, 35, 36, 37].

Abbreviations: AP, acute pancreatitis; apoB, apolipoprotein B; apoC‐III, apolipoprotein C‐III; ASCVD, atherosclerotic cardiovascular disease; FCS, familial chylomicronemia syndrome; HTG, hypertriglyceridemia; HTG‐AP, hypertriglyceridemia‐associated acute pancreatitis; IDL, intermediate‐density lipoprotein; LDL‐C, low‐density lipoprotein cholesterol; LPL, lipoprotein lipase; MASLD, metabolic dysfunction‐associated steatotic liver disease; MCS, multifactorial chylomicronemia syndrome; non‐HDL‐C, non‐high‐density lipoprotein cholesterol; PCSK9, proprotein convertase subtilisin/kexin Type 9; TG, triglycerides; TRL, triglyceride‐rich lipoprotein; VLDL, very‐low‐density lipoprotein.

This narrative review proposes a clinical framework for understanding and managing HTG according to phenotype and organ‐specific risk. It begins with the heterogeneous causes of HTG, with particular attention to the interaction between genetic susceptibility and acquired metabolic factors. The review also examines how disturbances in TRL metabolism contribute differently to pancreatic injury and ASCVD. On this basis, we discuss clinical phenotyping and diagnostic assessment, focusing on the distinction between pancreatitis‐predominant and ASCVD‐predominant HTG. Current and emerging treatment options are then evaluated in relation to TG severity, the presence of chylomicronemia, remnant lipoprotein burden, and the organ‐specific outcomes most relevant to the individual patient.

## 2 Etiology of HTG

HTG arises from the combined effects of genetic and acquired factors and, in most cases, reflects an imbalance among the production, lipolysis, and clearance of TRLs [8]. In the fasting state, very‐low‐density lipoproteins (VLDL) carry most circulating TG. After a meal, intestinal CMs and their remnants make a substantial contribution to the circulating TG pool [38, 39]. HTG therefore represents more than an isolated increase in plasma TG concentration. It reflects disturbed TRL metabolism, with changes in TRL particle number, size, composition, and circulating residence time.

Traditionally, HTG has been classified into primary (genetic) and secondary (acquired) forms [40]. In clinical practice, however, only a small proportion of patients have a monogenic disorder caused by biallelic pathogenic variants affecting the lipoprotein lipase (LPL) pathway, whereas most patients do not fit neatly into a single etiologic category [41]. For most patients, HTG reflects a continuous phenotype shaped by genetic background, metabolic status, lifestyle, and drug exposure. This spectrum‐based view of HTG helps explain its clinical heterogeneity. It also helps distinguish phenotypes dominated by chylomicronemia and AP risk from those characterized by TRL remnants, apoB particle burden, and residual ASCVD risk. Table 2 summarizes the etiologic stratification of HTG, with emphasis on factors associated with severe HTG and extreme HTG.

**TABLE 2:** Etiologic stratification of hypertriglyceridemia.

Primary category	Etiologic category	Representative entities or triggers	Typical TG phenotype	Key features
Abnormal lipoprotein metabolism and genetic susceptibility	Monogenic chylomicronemia	FCS	Persistent extreme HTG, typically with repeated fasting TG levels ≥11.3 mmol/L (≥1000 mg/dL), although historical FCS diagnostic scores have used repeated fasting TG levels >10 mmol/L (>885 mg/dL) as a clinical identification criterion; fasting chylomicronemia is characteristically present [42, 43].	Caused by biallelic pathogenic variants in genes involved in the LPL pathway, including LPL, APOC2, APOA5, GPIHBP1, and LMF1. Typically characterized by early onset, persistent chylomicronemia, and a high risk of HTG‐AP [43, 44, 45, 46].
Abnormal lipoprotein metabolism and genetic susceptibility	Multifactorial chylomicronemia	MCS	Recurrent, fluctuating, or persistent extreme HTG. TG levels may rise markedly when metabolic or other secondary factors are superimposed [35, 47].	The most common cause of chylomicronemia, especially in adults. It results from genetic susceptibility combined with secondary drivers such as obesity, diabetes, alcohol intake, pregnancy, TG‐raising medications, or dietary factors [35, 47, 48].
Secondary or precipitating factors	Metabolic and endocrine triggers	Obesity, metabolic syndrome, insulin resistance, diabetes mellitus, hypothyroidism [12, 36]	Usually mild‐to‐moderate HTG; may progress to severe or extreme HTG in the setting of poor metabolic control or underlying genetic susceptibility [12, 40].	Promote HTG by increasing VLDL production or impairing TG clearance. Poorly controlled diabetes is an important precipitating factor for MCS and HTG‐AP [20, 47, 49].
Secondary or precipitating factors	Exogenous and disease‐related triggers	Alcohol intake, excessive intake of dietary fat or simple carbohydrates, pregnancy, TG‐raising medications, kidney disease, and autoimmune diseases, such as SLE [2, 50, 51].	Variable severity; in genetically or metabolically susceptible individuals, these factors may precipitate severe extreme HTG [5, 50].	Usually act as triggering or amplifying factors. They may convert underlying genetic or metabolic susceptibility into clinically overt chylomicronemia, thereby increasing the risk of HTG‐AP [52, 53, 54].

*Note*: This table uses the operational terminology applied throughout this review. Severe HTG refers to fasting TG levels of 5.6–11.3 mmol/L (500–999 mg/dL), whereas extreme HTG, also termed chylomicronemia‐range HTG in recent consensus statements, refers to TG levels ≥11.3 mmol/L (≥1000 mg/dL). Chylomicronemia refers to the lipoprotein phenotype characterized by abnormal accumulation of circulating CMs and is not synonymous with all cases of severe HTG. Persistent chylomicronemia is defined as TG levels ≥11.3 mmol/L (≥1000 mg/dL) in more than half of the available measurements. The historical threshold of >10 mmol/L (>885 mg/dL), which has been incorporated into clinical scoring systems for the identification of FCS, is presented as a disease‐specific diagnostic clue and does not replace the operational classification applied throughout this review. TG thresholds are intended to support phenotypic description and risk stratification and should not be used in isolation to diagnose FCS or MCS. Differentiation between these conditions requires consideration of the persistence and variability of TG elevation, age at onset, secondary factors, clinical manifestations, response to conventional therapy, family history, and, when appropriate, genetic testing.

Abbreviations: APOA5, apolipoprotein A5; APOC2, apolipoprotein C2; FCS, familial chylomicronemia syndrome; GPIHBP1, glycosylphosphatidylinositol‐anchored high‐density lipoprotein‐binding protein 1; HTG, hypertriglyceridemia; HTG‐AP, hypertriglyceridemia‐associated acute pancreatitis; LMF1, lipase maturation factor 1; LPL, lipoprotein lipase; MCS, multifactorial chylomicronemia syndrome; SLE, systemic lupus erythematosus; TG, triglycerides; TRL, triglyceride‐rich lipoprotein; VLDL, very‐low‐density lipoprotein. For TG, 1 mmol/L is approximately equivalent to 88.6 mg/dL.

### 2.1 Genetic Architecture: Monogenic and Polygenic Susceptibility

Primary HTG includes rare monogenic disorders and more common polygenic forms [44]. The best‐characterized monogenic disorder is FCS, which is usually caused by biallelic pathogenic variants affecting genes in the LPL pathway. These genes include LPL, apolipoprotein C2 (APOC2), apolipoprotein A5 (APOA5), glycosylphosphatidylinositol‐anchored high‐density lipoprotein (HDL)‐binding protein 1 (GPIHBP1), and lipase maturation factor 1 (LMF1) [43]. These defects impair LPL‐mediated lipolysis of TRLs and reduce the clearance of CMs. This process leads to PC and extremely high TG levels [55, 56].

From a lipoprotein phenotype perspective, FCS is typically characterized by markedly elevated TG levels and a predominance of CMs. Plasma apoB levels are disproportionately low relative to TG levels, resulting in a high TG/apoB ratio [35, 43, 57]. This pattern suggests that the main abnormality is the accumulation of large TG‐rich particles, rather than a marked increase in the number of apoB‐containing particles. Because CMs are large particles, they are less able to penetrate an intact arterial intima. In addition, patients with FCS usually have a relatively low apoB particle burden. Therefore, their main clinical risks are recurrent abdominal pain and AP [5, 58]. FCS can therefore be viewed as a prototypical genetic form of pancreatitis‐predominant HTG.

Although FCS has a distinctive clinical presentation, it is exceedingly rare [46]. Most patients with extreme HTG do not carry biallelic pathogenic variants affecting the LPL pathway. Instead, they typically have polygenic or, less commonly, oligogenic susceptibility, in which multiple TG‐raising variants interact with metabolic and other secondary factors to increase TRL production and impair lipolysis and clearance [47, 59, 60]. In these patients, hepatic overproduction of VLDL often coexists with impaired peripheral TRL lipolysis and reduced remnant clearance. This combination produces a mixed lipoprotein phenotype characterized by elevated VLDL, TRL remnants, including intermediate‐density lipoproteins (IDL) and RC [8, 48].

The interaction between genetic susceptibility and secondary factors can lead to multifactorial chylomicronemia syndrome (MCS) [35, 61, 62]. Patients with MCS usually have higher apoB levels than patients with FCS. However, the apoB levels of the two groups can overlap substantially. Therefore, apoB alone cannot confirm or exclude FCS. Compared with FCS, MCS is more often associated with elevated non‐HDL cholesterol (non‐HDL‐C), MASLD, and other cardiometabolic abnormalities [63]. Therefore, MCS can show a phenotype that lies between pancreatitis‐ predominant HTG and ASCVD‐predominant HTG.

Beyond FCS and MCS, other inherited or genetically predisposed lipid disorders can also present with HTG or mixed dyslipidemia. These disorders include familial HTG, familial combined hyperlipidemia, familial dysbetalipoproteinemia, and inherited lipodystrophy. They involve different mechanisms, including abnormal VLDL composition or clearance, overproduction of apoB‐containing particles, impaired remnant clearance, and reduced lipid storage capacity in adipose tissue [44, 64, 65, 66]. Together, these disorders show that the genetic basis of HTG is not limited to a single pathway defect. Instead, different genetic and metabolic mechanisms can produce HTG or mixed dyslipidemic phenotypes.

In recent years, PC has gained attention as a phenotype‐oriented concept. According to the 2025 joint expert consensus from the National Lipid Association and the American Society for Preventive Cardiology, PC is defined as TG levels ≥1000 mg/dL (11.3 mmol/L) in more than half of measurements. Rather than replacing the etiologic classification of chylomicronemia into FCS and MCS, this framework provides a complementary approach based on the persistence of extreme TG elevation and the risk of pancreatitis. The consensus further identifies alarm features associated with a particularly high risk of AP, including recurrent TG‐induced AP, recurrent hospitalizations for severe abdominal pain without another identifiable cause, childhood pancreatitis, a family history of TG‐induced pancreatitis, and postheparin LPL activity <20% of the normal value [5]. This phenotype‐oriented approach may facilitate the identification and risk stratification of high‐risk patients, including those in whom genetic testing is unavailable or inconclusive, and help guide intensive nutritional and pharmacological management, genetic evaluation, and consideration of emerging targeted therapies [67].

### 2.2 Acquired Modifiers of TG Metabolism

Acquired or secondary factors can raise TG levels by promoting hepatic VLDL production, increasing the delivery of FFAs to the liver, inhibiting LPL activity, or impairing the clearance of TRL remnants. In most patients, metabolic disturbances are the main drivers of secondary HTG and often interact with diet, alcohol use, physiological states, and drug exposure [2, 68].

#### 2.2.1 Cardiometabolic and Endocrine Drivers

Insulin resistance is a central metabolic driver of common HTG. It enhances lipolysis in adipose tissue and increases the delivery of FFAs to the liver. This process promotes hepatic TG synthesis and VLDL secretion [69]. Insulin resistance can also reduce LPL activity and impair TRL lipolysis and remnant clearance. Therefore, insulin resistance promotes the development and persistence of HTG by increasing TG production and reducing TRL clearance [70, 71].

Obesity, type 2 diabetes, and metabolic syndrome are the most common clinical backgrounds of HTG [72, 73]. Poor glycemic control can further increase VLDL production and impair TRL clearance. This process can lead to marked TG elevation. MASLD is also closely linked to HTG. This association is driven by shared metabolic abnormalities, including increased hepatic de novo lipogenesis (DNL), insulin resistance, increased fatty acid flux, and VLDL overproduction [74, 75, 76]. These metabolic abnormalities often lead to persistent elevations in VLDL and TRL remnants. They may also contribute to residual ASCVD risk.

Cardiovascular–kidney–metabolic syndrome provides a more integrated clinical framework for understanding HTG [77]. Abdominal obesity, insulin resistance, diabetes, high TG, low high‐density lipoprotein cholesterol (HDL‐C), hypertension, albuminuria, and chronic kidney disease (CKD) often coexist. These conditions can amplify one another through abnormalities in lipid metabolism, inflammation, endothelial dysfunction, and renal metabolic regulation [78, 79]. Therefore, common HTG is better understood not as an isolated lipid disorder, but as a manifestation of systemic cardiometabolic dysfunction reflected in disturbed TRL metabolism.

Endocrine disorders are also important acquired modifiers of HTG. Hypothyroidism can reduce lipoprotein metabolism and impair remnant clearance. Cushing syndrome and chronic glucocorticoid exposure can raise TG levels by increasing insulin resistance, fatty acid mobilization, and VLDL production. Acromegaly, polycystic ovary syndrome, and other endocrine conditions associated with insulin resistance may also worsen HTG [80, 81, 82]. In patients with new‐onset, worsening, or refractory HTG, a systematic assessment of glucose metabolism, thyroid function, kidney function, liver function, urine protein, and relevant endocrine conditions is an important part of etiologic evaluation [83].

#### 2.2.2 Dietary, Alcohol‐Related, and Physiological Modifiers

Dietary composition is one of the most important and modifiable acquired factors in HTG. Excess intake of refined carbohydrates and fructose can stimulate hepatic DNL. This process increases TG synthesis and promotes VLDL secretion [84]. Alcohol use can also raise TG levels by stimulating hepatic TG synthesis and VLDL secretion and by impairing TRL clearance [85, 86]. These effects are generally dose dependent. In individuals with insulin resistance, diabetes, or underlying genetic susceptibility, alcohol consumption may precipitate severe or extreme HTG and thereby increase the risk of AP [5, 87].

Pregnancy represents a distinct physiological state associated with substantial changes in lipid metabolism. Increases in estrogen and placental hormones, together with pregnancy‐related insulin resistance, promote hepatic VLDL production and reduce LPL‐mediated TG clearance. Consequently, TG levels rise physiologically during pregnancy, particularly in the third trimester, but generally remain below the severe range in women without underlying susceptibility. In women with genetic or metabolic susceptibility, however, pregnancy may precipitate severe HTG, extreme HTG, or even AP [88, 89, 90]. Therefore, among women of reproductive age or in pregnant patients, it is important to identify a prior history of TG elevation, family history, metabolic risk factors, and drug exposure. This approach may help prevent complications related to pregnancy‐associated HTG.

Other nutritional and lifestyle factors can also worsen HTG. These factors include sedentary behavior, weight gain, and excessive energy intake [12, 40]. Total parenteral nutrition containing lipid emulsions can also raise TG levels, especially when lipid tolerance is reduced or serum TG is not adequately monitored [91, 92].

#### 2.2.3 Drug‐Induced HTG

Drug‐induced HTG is an often overlooked but modifiable cause of TG elevation. Different drugs can raise TG levels through several mechanisms. These mechanisms include weight gain and insulin resistance, increased hepatic VLDL production, inhibition of LPL activity, impaired clearance of TRL remnants, and increased exogenous lipid load [12, 93]. The degree of drug‐related TG elevation often depends on the patient's baseline metabolic status and underlying susceptibility. In patients with diabetes, obesity, insulin resistance, pregnancy, or a history of HTG, drug exposure may trigger marked TG elevation, especially in the presence of polygeinc susceptibility or monogenic defects affecting TG metabolism [45, 51].

Atypical antipsychotic drugs, such as olanzapine, can cause HTG through weight gain and insulin resistance [94, 95, 96]. Oral estrogen preparations and tamoxifen can increase VLDL‐TG synthesis and secretion [97, 98]. Retinoids, including isotretinoin and bexarotene, can increase VLDL production and impair TRL clearance, leading to mild‐to‐moderate or even severe TG elevation [99, 100]. Traditional β‐blockers, such as propranolol and atenolol, and thiazide diuretics, such as hydrochlorothiazide, are also associated with TG elevation [101]. The effect of thiazide diuretics may involve hypokalemia, abnormal glucose metabolism, and reduced insulin sensitivity [102]. The metabolic effects of β‐blockers vary by drug type. Traditional β‐blockers can raise TG levels and lower HDL‐C by inhibiting LPL activity. In contrast, newer vasodilating β‐blockers, such as carvedilol and nebivolol, have a more neutral effect on lipid metabolism. Glucocorticoids can affect TRL metabolism through glucocorticoid receptor‐dependent mechanisms, including induction of angiopoietin‐like protein 4 (ANGPTL4) expression and inhibition of LPL activity [103, 104].

Immunomodulatory or immunosuppressive agents are also important drug‐related causes of HTG. These agents include cyclosporine, tacrolimus, sirolimus, and interferons. They can cause HTG by affecting hepatic lipoprotein production, insulin sensitivity, or lipoprotein clearance [105, 106, 107]. Certain human immunodeficiency virus (HIV) protease inhibitors can also induce insulin resistance, fat redistribution, and increased VLDL production. These effects are important contributors to HTG and increased ASCVD risk in people living with HIV [108, 109]. Cancer therapy‐related drugs, such as L‐asparaginase can also cause marked HTG. Some patients may even develop AP [110, 111]. Long‐term or high‐dose propofol infusion, parenteral nutrition containing lipid emulsions, and bile acid sequestrants may also worsen TG elevation in specific clinical settings [91, 112, 113].

In patients with diabetes, obesity, insulin resistance, or underlying genetic susceptibility, drug‐related TG elevation may add to pre‐existing metabolic abnormalities. This additive effect may precipitate severe HTG and related complications [114, 115, 116]. Therefore, a systematic review of medication history is essential when evaluating HTG. Clinical management should balance the benefits of treating the primary disease against potential adverse metabolic effects. Based on this assessment, clinicians may consider alternative drugs, dose adjustment, TG monitoring, or preventive intervention.

When these acquired factors act on a background of genetic susceptibility and raise TG to extremely high levels, the risk of AP can increase substantially. This risk may be further amplified by poor glycemic control, alcohol use, pregnancy, kidney disease, or exposure to TG‐raising drugs. These observations suggest that acquired factors not only influence the degree of TG elevation but may also shape whether patients shift toward a pancreatitis‐predominant or ASCVD‐predominant phenotype.

## 3 Pathophysiological Basis of HTG and Mechanisms of Tissue Injury

HTG is a disorder of TRL metabolism. Its core features include increased TRL production, impaired lipolysis, and reduced clearance of remnant particles [117]. Recent dyslipidemia guidelines and expert consensus statements increasingly frame the management of HTG within two major clinical contexts: prevention of AP and reduction of ASCVD risk. In patients with extreme HTG, particularly when chylomicronemia is persistent, prevention of AP becomes the immediate clinical priority. In persistent mild‐to‐moderate HTG, TRL remnants, RC, non‐HDL‐C, and apoB‐containing particle burden are more closely linked to long‐term residual ASCVD risk [12, 118].

The relative contribution of each mechanism varies across HTG phenotypes. Extreme HTG accompanied by substantial CM accumulation is more likely to cause pancreatic injury. Persistent increases in TRL remnants and apoB‐containing particle burden are more likely to promote chronic vascular injury [30, 119]. In severe or persistent HTG, abnormal accumulation of circulating TRLs and their remnants can contribute to mulitiple pathophysiological processes. These processes include FFA‐mediated lipotoxicity, mitochondrial dysfunction, oxidative stress, ER stress, disturbed Ca^2^^+^ homeostasis, regulated cell death, microcirculatory impairment, intestinal barrier disruption, and activation of inflammatory signaling. Together, these mechanisms link abnormal lipoprotein metabolism to local tissue injury, systemic inflammatory responses, and remote organ injury [120, 121, 122, 123] (Figure 1).

**FIGURE 1:**
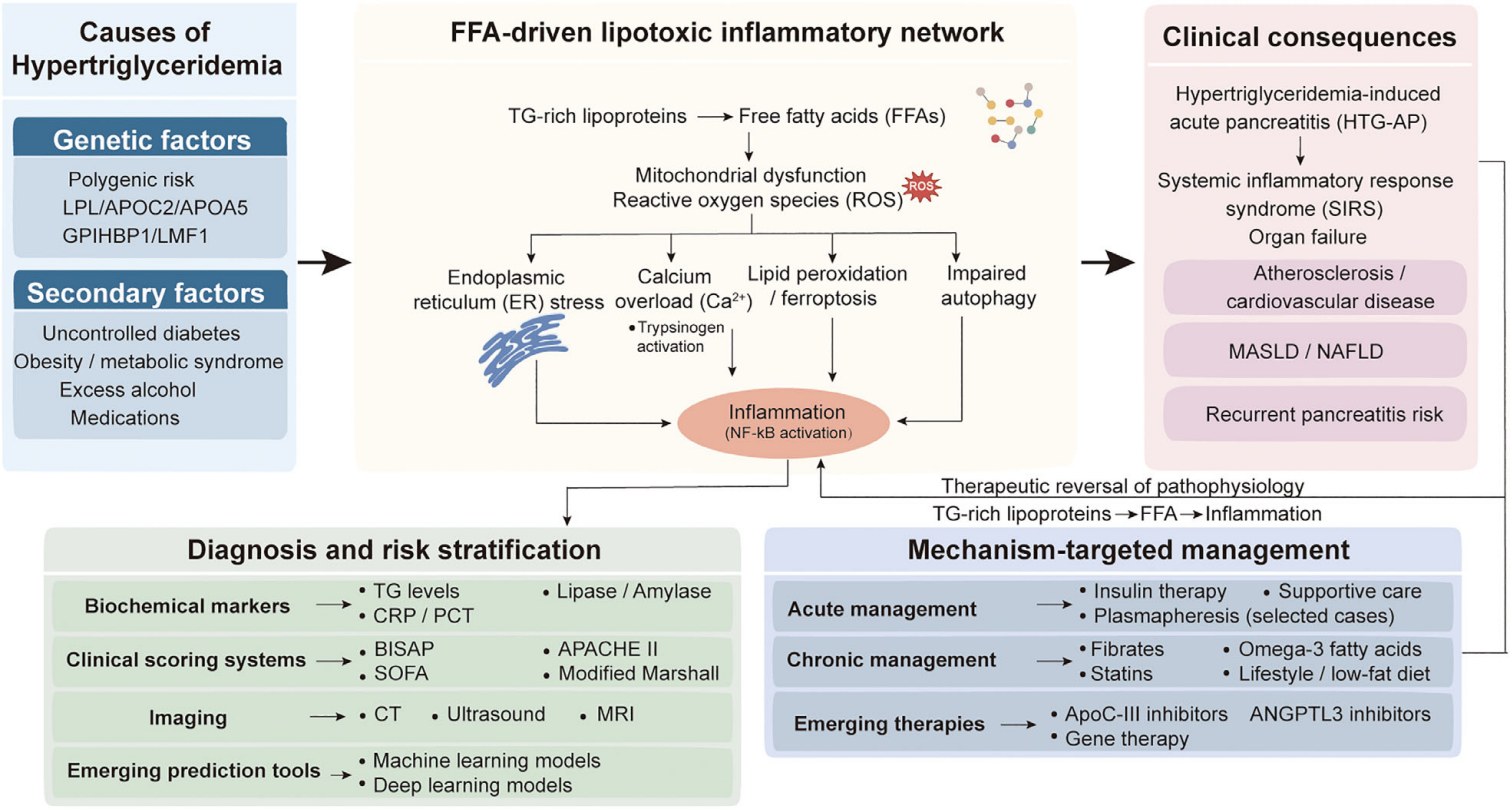
An integrated pathophysiological and clinical framework of hypertriglyceridemia. HTG arises from genetic and secondary factors and promotes FFA‐driven lipotoxic inflammation through mitochondrial dysfunction, oxidative stress, ER stress, calcium overload, ferroptosis, and impaired autophagy. This inflammatory network links HTG to AP, systemic complications, and cardiometabolic disease. Diagnosis, risk stratification, and mechanism‐targeted management strategies are summarized in the lower panels.

The strength of clinical evidence for HTG‐related tissue injury mechanisms varies across pathways. The clinical association between severe HTG, extreme HTG, and AP risk is well established [7]. The relationships of TRL remnants, RC, non‐HDL‐C, and apoB‐containing particle burden with ASCVD risk are also supported by human epidemiological, genetic, and clinical evidence. Mechanisms such as FFA‐mediated lipotoxicity, microcirculatory impairment, oxidative stress, ER stress, disturbed Ca^2^^+^ homeostasis, and activation of nuclear factor‐κB (NF‐κB) and the NLR family pyrin domain‐containing 3 (NLRP3) inflammasome are supported by a combination of human observational studies, diseased tissue samples, and experimental models. These mechanisms can be viewed as having translational relevance [121]. Other proposed mechanisms, including ferroptosis, impaired autophagy, neutrophil extracellular trap (NET) formation, gut microbiota dysbiosis, and an imbalance between T helper 17 (Th17) cells and regulatory T (Treg) cells, are supported mainly by evidence from animal models, in vitro experiments, or limited human association studies [124]. At present, these mechanisms should be regarded as emerging rather than clinically established. Therefore, this review discusses these mechanisms as potential amplifying pathways in HTG‐related tissue injury, rather than as fully established clinical causal pathways.

### 3.1 Determinants of TG Elevation: Imbalance Between Production and Clearance

Circulating TG is mainly transported by TRLs, including liver‐derived VLDL, intestine‐derived CMs, and their remnant particles. In the fasting state, plasma TG is mainly carried by VLDL. In the postprandial state, CMs and their remnants make a greater contribution to the circulating TG load [125, 126]. Therefore, plasma TG levels reflect the dynamic balance among TRL production, lipolysis, and clearance. In most cases, HTG does not result from a single defect. Instead, It reflects the combined effects of increased hepatic VLDL production, impaired peripheral TRL lipolysis, and reduced remnant particle clearance [127].

Hepatic VLDL overproduction is an important driver of polygenic and acquired HTG [128]. Insulin resistance in adipose tissue weakens the antilipolytic effect of insulin. This change increases the delivery of FFAs to the liver and provides substrate for hepatic TG synthesis [129]. Excess intake of refined carbohydrates and fructose, metabolic syndrome, and MASLD can further increase hepatic lipid burden by activating hepatic DNL. Sterol regulatory element‐binding protein 1c and carbohydrate‐responsive element‐binding protein are key transcriptional regulators of this process [130, 131]. In selective hepatic insulin resistance, insulin‐mediated suppression of hepatic glucose production is impaired, whereas lipogenic signaling remains relatively preserved. This imbalance promotes hepatic TG synthesis and VLDL secretion, thereby increasing the circulating VLDL‐TG burden [132, 133, 134].

Impaired TRL clearance is another key mechanism in HTG. Peripheral TRL metabolism mainly depends on LPL‐mediated lipolysis. LPL activity is regulated by several apolipoproteins, endothelial binding proteins, and circulating inhibitory factors [135]. In monogenic HTG, functional defects in genes such as LPL, APOC2, APOA5, GPIHBP1, and LMF1 can severely impair TRL lipolysis. These defects cause PC and extremely high TG levels [136, 137]. In polygenic HTG, the cumulative effects of multiple common variants and some rare variants can reduce the efficiency of TRL clearance. Under conditions of insulin resistance and metabolic stress, LPL inhibitors such as apoC‐III, ANGPTL3, and ANGPTL4 can further impair TRL lipolysis and contribute to delayed remnant clearance [138, 139, 140].

These metabolic pathways not only influence the degree of TG elevation but also shape the clinical phenotype. Patients whose disease is driven mainly by severe LPL pathway defects or impaired CM clearance are more likely to develop AP. Patients characterized by VLDL overproduction, impaired remnant clearance, and increased apoB‐containing particle burden are more likely to develop arterial lipid retention, vascular inflammation, and ASCVD progression.

### 3.2 Lipid Overload and Lipotoxicity as Initiating Events

Lipid overload is an early event in HTG‐related tissue injury. As circulating TRLs increase markedly, LPL‐mediated hydrolysis can release large amounts of FFAs. When FFA generation exceeds the buffering capacity of albumin binding and cellular metabolism, unbound FFAs and toxic lipid intermediates can induce lipotoxic injury [141]. Although FFAs are important energy substrates under physiological conditions, their excessive accumulation can disrupt cell membrane structure, mitochondrial function, and intracellular signaling. It can also promote oxidative stress, ER stress, and inflammatory responses [17, 142, 143].

This process is particularly important in pancreatitis‐ predominant HTG. Severe chylomicronemia can markedly increase TRLs and CMs in the pancreatic microcirculation. Local pancreatic lipase‐mediated hydrolysis then releases high concentrations of FFAs. These FFAs can directly injure acinar cells and vascular endothelial cells, leading to lipotoxicity, microcirculatory impairment, acinar cell injury, and activation of inflammatory cascades [144]. In ASCVD‐ predominant HTG, lipid‐related injury is more closely related to the retention of TRL remnants and apoB‐containing particles in the arterial wall. Smaller VLDL remnants, IDL, and other remnant particles can enter the arterial intima and deliver cholesterol to the vessel wall. Cholesterol deposition, oxidative modification, and inflammatory activation can then promote endothelial dysfunction, monocyte recruitment, foam cell formation, and plaque progression [145]. Therefore, lipid‐mediated injury in HTG shows marked tissue specificity [15]. In the pancreas, it mainly manifests as acute lipotoxic and inflammatory injury. In the vasculature, it more often manifests as chronic cholesterol deposition and inflammatory atherosclerosis.

### 3.3 Mitochondrial Dysfunction and Oxidative Stress

Mitochondrial dysfunction is a key hub that links lipid overload to tissue injury. After entering cells, excess FFAs can increase the burden of mitochondrial β‐oxidation and interfere with electron transport chain function. This process leads to excessive production of reactive oxygen species (ROS). ROS accumulation further damages mitochondrial DNA, proteins, and lipids, thereby disrupting intracellular homeostasis [146, 147]. Mitochondrial injury and ROS generation can form a positive feedback loop that progressively amplifies metabolic stress and downstream pathological signaling.

Different fatty acid species may induce distinct patterns of cellular stress. Under some experimental conditions, unsaturated fatty acids are more closely associated with mitochondrial dysfunction, increased intracellular Ca^2^^+^ levels, and inflammatory injury. Saturated fatty acids are more likely to induce ER stress [122, 148]. These findings still need further validation in human disease settings. However, they suggest that HTG‐related tissue injury is influenced not only by TG levels but also by fatty acid composition, the local rate of lipolysis, and the local metabolic environment.

Through mitochondrial injury and oxidative stress, HTG can promote both acute pancreatic injury and chronic vascular injury. In the pancreas, ROS can induce acinar cell injury, abnormal zymogen activation, enhanced inflammatory signaling, and cell death [149, 150]. In the vasculature, oxidative stress can cause endothelial dysfunction, monocyte recruitment, vascular inflammation, and plaque instability [151, 152, 153]. Therefore, oxidative stress is an important shared mechanism linking pancreatitis‐predominant HTG and ASCVD‐predominant HTG, although its tissue localization and clinical consequences differ.

### 3.4 ER Stress and Disruption of Calcium Homeostasis

ER stress is an important downstream response to lipid overload and oxidative stress [154, 155]. When misfolded or unfolded proteins accumulate in the ER, cells activate the unfolded protein response (UPR) to restore protein folding and cellular homeostasis. However, when ER stress persists and the UPR fails to restore homeostasis, this response can lead to cellular dysfunction, inflammatory activation, and apoptosis [156, 157].

In HTG‐AP, ER stress is closely linked to disturbed Ca^2^^+^ homeostasis. Excess FFAs and ROS can impair ER calcium storage, leading to sustained elevation of cytosolic Ca^2^^+^ levels [158]. Ca^2^^+^ overload is an important upstream event that links metabolic stress to abnormal zymogen activation and inflammatory signaling [144, 159]. In pancreatic acinar cells, sustained Ca^2^^+^ elevation can promote premature trypsinogen activation, which is one of the hallmark initiating events in AP [160]. Ca^2^^+^ overload can also activate inflammatory pathways such as NF‐κB, thereby converting early cellular injury into local and systemic inflammatory responses [161, 162, 163].

This mechanism further helps explain why extreme HTG accompanied by substantial CM accumulation predisposes to acute pancreatic injury. In ASCVD‐predominant HTG, ER stress and disturbed Ca^2^^+^ homeostasis contribute to vascular cell stress responses. Together with oxidative stress, lipid accumulation, and inflammatory signaling, they promote endothelial dysfunction, vascular smooth muscle cell phenotypic switching, and macrophage foam cell formation.

### 3.5 Regulated Cell Death, Autophagy Impairment, and NETs

Beyond classical lipotoxicity and organelle stress, regulated cell death, impaired autophagy, and NET formation may contribute to HTG‐related tissue injury and inflammatory amplification. Current evidence for these mechanisms comes mainly from experimental studies, and their causal roles in humans still need further validation. Ferroptosis is a form of regulated cell death characterized by iron‐dependent lipid peroxidation. It can be driven by excessive ROS generation, increased lipid peroxidation, and impaired antioxidant defense [164]. In HTG‐related tissue injury, excess FFAs and oxidative stress may promote the accumulation of lipid peroxides. This process may further amplify inflammatory responses through the release of damage‐associated molecular patterns (DAMPs) [165].

Autophagy has a protective role in clearing damaged organelles, misfolded proteins, and byproducts of lipid metabolism [166]. However, under conditions of lipid overload and persistent oxidative stress, autophagic flux may become impaired [167]. Impaired autophagy can lead to the accumulation of dysfunctional mitochondria, toxic protein aggregates, and lipid intermediates. These changes further increase ROS generation, ER stress, and inflammatory responses [168, 169]. In HTG‐AP, impaired autophagy may promote pancreatic injury by disrupting zymogen granule processing, facilitating abnormal trypsinogen activation, and impairing acinar cell homeostasis [170, 171, 172]. In atherosclerosis, impaired macrophage autophagy can reduce cholesteryl ester mobilization and ATP‐binding cassette transporter A1‐dependent cholesterol efflux. This process promotes foam cell formation, inflammatory amplification, and plaque instability [173, 174, 175].

NETs represent another potential mechanism of inflammatory amplification in HTG [176, 177]. HTG‐related metabolic disturbances, activation of toll‐like receptor 4 (TLR4) signaling, and release of DAMPs such as high mobility group box 1 may promote NET formation or enhance NET‐mediated proinflammatory effects [178, 179]. In HTG‐AP, NETs may exacerbate local pancreatic inflammation, immune cell infiltration, and tissue injury [124]. In the vascular environment, NETs may contribute to thromboinflammation and atherosclerosis progression by inducing endothelial cell activation, enhancing a procoagulant phenotype, and promoting the aggregation of platelets and coagulation factors [180, 181]. Together, ferroptosis, impaired autophagy, and NET formation provide new mechanistic insights into how metabolic abnormality in HTG may translate into inflammatory tissue injury. Their causal roles in human HTG‐related diseases still require further validation.

### 3.6 Microcirculatory Dysfunction and Gut Barrier Impairment in HTG‐AP

In severe HTG, especially in the setting of chylomicronemia, microcirculatory impairment is an important mechanism in the onset and progression of HTG‐AP [124]. Large amounts of CMs and large VLDL particles can increase plasma viscosity and reduce pancreatic microvascular perfusion. These changes can lead to local ischemia, hypoxia, and acidosis [182]. In addition, increased thromboxane A_2_ (TXA_2_) production and an imbalance between TXA_2_ and prostacyclin (PGI_2_) in HTG can induce vasoconstriction, platelet activation, and microthrombus formation. These effects further aggravate pancreatic microcirculatory impairment [121].

Intestinal barrier injury may serve as an important amplifying step in the progression of HTG‐AP from local pancreatic inflammation to a systemic inflammatory response. AP‐related hypoperfusion, intestinal mucosal ischemia and hypoxia, and metabolic stress can disrupt intestinal epithelial integrity and promote gut microbiota dysbiosis [183, 184, 185]. HTG‐related lipid overload may also alter the gut microbiota [186]. Severe HTG and high‐fat metabolic states are often associated with altered gut microbiota composition. Reported alterations include reductions in beneficial bacteria, such as Bifidobacterium and Lactobacillus, and increases in potentially pathogenic bacteria, such as Enterobacteriaceae and other Gram‐negative bacteria [187]. These changes may be accompanied by reduced production of short‐chain fatty acids (SCFAs). SCFAs play an important role in maintaining intestinal epithelial barrier integrity and immune homeostasis [188, 189].

Gut microbiota dysbiosis and barrier disruption can increase intestinal permeability. This change allows bacteria and their products, especially lipopolysaccharide (LPS), to enter the portal and systemic circulation, contributing to microbial translocation and endotoxemia [190, 191]. This process provides a mechanistic link between HTG‐related metabolic disturbances and systemic inflammatory responses [192].

Translocated microbial components can act as pathogen‐associated molecular patterns. They activate pattern‐recognition receptors, especially the TLR4/NF‐κB pathway, and promote the release of proinflammatory cytokines such as tumor necrosis factor‐α (TNF‐α) and interleukin‐6 (IL‐6) [193, 194]. In addition, HTG‐AP and intestinal barrier injury are also associated with activation of the NLRP3 inflammasome. NLRP3 activation promotes Caspase‐1‐mediated maturation and secretion of IL‐1β and IL‐18, thereby amplifying local and systemic inflammatory cascades [195]. Evidence also suggests that gut microbiota dysbiosis may further disrupt intestinal immune homeostasis by disturbing the Th17/Treg balance, promoting bacterial translocation and systemic complications [196, 197, 198].

Microcirculatory impairment, intestinal barrier disruption, and gut microbiota dysbiosis may therefore constitute interconnected amplifying mechanisms in the progression of HTG‐AP from local pancreatic injury to SIRS, organ failure, and distant‐organ injury. More broadly, microbiome signatures and microbiota‐directed interventions are increasingly being investigated as potential diagnostic, prognostic, and therapeutic tools in other disease fields, particularly oncology [199]. These advances highlight the potential translational value of microbiome research, although whether similar approaches could improve risk stratification or treatment in HTG‐AP remains unknown. The causal contributions, temporal sequence, and therapeutic targetability of microcirculatory and gut‐derived mechanisms in human HTG‐AP therefore require further validation.

### 3.7 Inflammatory Signaling in HTG‐Related Tissue Injury

Activation of inflammatory signaling is a convergent downstream pathway in HTG‐related tissue injury. During the onset and progression of AP, upstream mechanisms such as lipotoxicity, oxidative stress, ER stress, disturbed Ca^2^^+^ homeostasis, regulated cell death, microcirculatory impairment, and intestinal barrier disruption may collectively promote inflammatory cascades. HTG amplifies these inflammatory processes by increasing lipid substrate burden, promoting FFA release, and aggravating cellular stress [171].

NF‐κB is one of the central transcriptional regulators of HTG‐related inflammatory responses [200, 201]. FFAs, ROS, DAMPs, and LPS can all activate NF‐κB signaling, thereby promoting the expression of TNF‐α, IL‐1β, IL‐6, and chemokines. Intestinal barrier injury and gut microbiota dysbiosis can enhance TLR4/NF‐κB signaling and contribute to NLRP3 inflammasome activation by facilitating the entry of microbial products such as LPS into the circulation [202].

In HTG‐AP, these pathways may drive the expansion of local pancreatic inflammation into a systemic inflammatory response. They may also contribute to distant organ complications, including lung injury, kidney injury, and circulatory dysfunction. In ASCVD‐predominant HTG, persistent low‐grade inflammation can promote endothelial dysfunction, monocyte recruitment, foam cell formation, and plaque progression [203].

### 3.8 Integrated Mechanisms and Therapeutic Implications

HTG‐related tissue injury is not driven by a single mechanism. Instead, it reflects an interconnected mechanistic network involving disturbed TRL metabolism, lipid overload, FFA‐mediated lipotoxicity, organelle stress, disturbed Ca^2^^+^ homeostasis, regulated cell death, microcirculatory impairment, intestinal barrier disruption, and activation of inflammatory signaling [204]. In severe‐to‐extreme HTG, particularly when chylomicronemia is present, the accumulation of CMs and large TRLs, local FFA release, increased plasma viscosity, and pancreatic microcirculatory impairment collectively promote the development of HTG‐AP [205]. In persistent mild‐to‐moderate HTG, the accumulation of VLDL, TRL remnants, and other apoB‐containing particles promotes ASCVD progression through arterial wall retention, cholesterol deposition, endothelial dysfunction, and chronic inflammation [206].

This mechanistic divergence further supports phenotype‐based assessment and mechanism‐guided intervention in HTG. In pancreatitis‐predominant HTG, mechanism‐guided intervention focuses on lowering extremely high TG levels and CM burden, improving TRL lipolysis and clearance, correcting acquired triggers, and limiting lipotoxicity, inflammatory amplification, and acinar cell injury. In ASCVD‐predominant HTG, greater attention should be given to apoB‐containing particle burden, non‐HDL‐C, RC, remnant particle retention, and the associated endothelial dysfunction and chronic vascular inflammation. Recent progress in targeted therapies directed at targets such as apoC‐III and ANGPTL3 has created new possibilities for mechanism‐ and phenotype‐based precision intervention in HTG [207, 208].

Mechanistic research on HTG needs to move beyond explaining why TG levels rise and clarify how different TRL phenotypes drive organ‐specific injury. Integrating TG levels and their temporal changes, chylomicronemia status, TRL remnants and RC, apoB particle burden, metabolic background, and clinical events such as AP and ASCVD may help build a unified framework that connects etiology, pathophysiology, risk stratification, and treatment selection.

## 4 Clinical Consequences of HTG

The clinical consequences of HTG are highly heterogeneous. The major clinical outcomes include acute and potentially life‐threatening HTG‐AP, long‐term cumulative ASCVD, and a range of metabolic and systemic complications. These outcomes are largely determined by the degree and duration of TG elevation, the burden of CMs, VLDL, TRL remnants, and the other apoB‐ containing particles, and the combined effects of genetic and secondary factors.

### 4.1 ASCVD

ASCVD is one of the most important long‐term clinical consequences of HTG [118]. It is commonly observed in patients with persistent elevations in VLDL and TRL remnants. Whereas the predominant immediate concern in extreme HTG with marked chylomicronemia is AP, the principal pathogenic factor in the ASCVD‐predominant HTG phenotype is not the direct toxicity of TG itself. Rather, ASCVD risk is driven primarily by an increased burden of cholesterol‐enriched TRL remnants and other atherogenic apoB‐containing particles.

The role of TG in ASCVD has long been debated [8]. This debate partly reflects the substantial biological variability of TG levels, which are influenced by fasting and postprandial states [209]. It also reflects the close association of HTG with cardiometabolic risk factors such as obesity, insulin resistance, type 2 diabetes, and metabolic syndrome [210, 211]. Current evidence suggests that TG concentration itself is more likely to be a marker of disturbed TRL metabolism and increased remnant lipoprotein burden than an isolated primary atherogenic factor. The association between HTG and ASCVD risk is mainly mediated by TRLs and their cholesterol‐rich remnant particles, with this atherogenic burden reflected especially by increased RC and apoB particle number [212, 213].

Epidemiological studies have shown that elevated TG levels are associated with increased risks of coronary heart disease, ischemic stroke, and cardiovascular mortality in both fasting and nonfasting states [214, 215, 216]. However, this association is often attenuated after adjustment for low‐density lipoprotein cholesterol (LDL‐C), non‐HDL‐C, apoB, and other metabolic risk factors [217]. This observation does not negate the clinical importance of HTG. Rather, it suggests that elevated TG represents an integrated marker of atherogenic lipoprotein particle burden and cardiometabolic abnormalities. Accordingly, ASCVD risk assessment in patients with HTG requires a broader framework beyond the absolute TG concentration, incorporating non‐HDL‐C, apoB, LDL‐C, RC, diabetes status, kidney function, and overall cardiovascular risk profile.

From a pathophysiological perspective, TRL remnant particles have clear atherogenic potential [218]. Unlike large CMs, smaller VLDL remnants, IDL, and other small TRL remnants can penetrate the arterial intima and become retained within the vessel wall [219, 220]. These particles are apoB‐containing lipoproteins. Each particle can carry a relatively large cholesterol load and can be taken up by macrophages, thereby promoting foam cell formation and plaque progression [221, 222]. Unlike low‐density lipoprotein (LDL), remnant particles may be more readily taken up by macrophages and may promote foam cell formation without requiring extensive oxidative modification. This property further increases their atherogenic potential.

Genetic studies further support a causal role of TRL‐related pathways in ASCVD [223]. Multiple Mendelian randomization studies and human genetic studies have shown that variants affecting TRL clearance, VLDL metabolism, or the LPL regulatory network are associated with changes in ASCVD risk. These variants include those involving pathways related to LPL, apolipoprotein C3 (APOC3), ANGPTL3, and ANGPTL4 [140, 224, 225, 226, 227, 228]. These genetic effects are more likely to be mediated by changes in RC, apoB‐ containing particle number, and other atherogenic lipoprotein components than by changes in TG levels alone [119, 229].

ASCVD risk differs substantially across HTG phenotypes. Polygenic and acquired HTG are usually accompanied by hepatic VLDL overproduction, impaired clearance of TRL remnants, increased RC, reduced HDL‐C, insulin resistance, and increased apoB particle burden. Therefore, these patients may have substantial residual ASCVD risk even when LDL‐C is well controlled [230, 231]. In contrast, monogenic chylomicronemia, such as FCS, is characterized by extremely high TG levels, but its apoB particle burden is usually relatively low. Large CMs are also less able to enter the arterial intima. Therefore, ASCVD risk does not necessarily increase in proportion to the degree of TG elevation. This distinction suggests that the type, number, and cholesterol content of lipoprotein particles are more important determinants of atherogenicity than the absolute TG concentration [50].

Current epidemiological, genetic, and clinical evidence collectively indicates that HTG‐related ASCVD risk mainly reflects an increased burden of TRL remnant particles, RC, and apoB‐ containing lipoprotein particles, rather than isolated toxicity of the TG molecule itself [8, 232]. In ASCVD‐predominant HTG, TG is best interpreted as a clinical marker of increased cardiovascular risk and disturbed TRL metabolism. Accordingly, ASCVD risk stratification and treatment intensity rely on a broader lipid and risk framework that includes apoB, non‐HDL‐C, RC, LDL‐C, and overall cardiovascular risk [12, 233].

### 4.2 HTG‐AP

Unlike its chronic cumulative effect on ASCVD risk, HTG is a recognized cause of AP. It is commonly considered one of the major causes of AP after gallstone disease and alcohol use [234]. HTG‐AP accounts for approximately 2–5% of all AP cases [235]. However, this proportion may reach 10–20% in some Asian populations [23, 236]. These observations suggest that HTG‐AP has an important and potentially underestimated clinical burden, particularly among individuals with severe or extreme HTG, especially when chylomicronemia is present or persistent, and among those with diabetes, obesity, or underlying genetic susceptibility.

The relationship between TG levels and AP risk is dose related, but there is no single threshold that is either completely safe or invariably pathogenic. AP risk increases progressively as TG levels rise. TG levels above approximately 5.6 mmol/L (500 mg/dL) are generally considered clinically relevant for AP risk assessment [12, 237]. When TG levels reach or exceed 11.3 mmol/L (1000 mg/dL), chylomicronemia is often present, and AP risk increases further [1, 238]. HTG‐AP does not occur only in patients with extremely high TG levels. In the presence of secondary triggers, such as uncontrolled diabetes, alcohol use, pregnancy, obesity, genetic susceptibility, or exposure to TG‐raising drugs, clinically significant AP may occur even when TG levels are below 11.3 mmol/L (1000 mg/dL) [239].

The development of HTG‐AP is closely linked to chylomicronemia, lipotoxicity, and pancreatic microcirculatory impairment. In extreme HTG accompanied by chylomicronemia, large numbers of CMs and large VLDL particles accumulate in plasma. These particles increase plasma viscosity and reduce pancreatic capillary perfusion. As the largest TRL particles, CMs may contribute to capillary obstruction, local stasis, ischemia, and acidosis within the pancreatic microcirculation. This microenvironment not only aggravates microcirculatory impairment but also provides abundant substrate for pancreatic lipase‐mediated local TRL hydrolysis. Large amounts of FFAs are released within the pancreatic capillary bed. When FFA generation exceeds the binding capacity of albumin, unbound FFAs can directly injure acinar cells and vascular endothelial cells. They can also induce endothelial dysfunction, oxidative stress, disturbed Ca^2^^+^ homeostasis, and cell necrosis [240, 241].

Local injury further amplifies the inflammatory response. Mitochondrial dysfunction, ER stress, premature trypsinogen activation, and activation of NF‐κB and the NLRP3 inflammasome can drive progression from local pancreatic injury to a systemic inflammatory response. In severe cases, HTG‐AP may be accompanied by pancreatic necrosis, vascular leakage, SIRS, persistent organ dysfunction, and distant organ injury [242]. Compared with gallstone‐ or alcohol‐related AP, some studies suggest that patients with HTG‐AP may be more likely to develop severe disease, organ failure, or recurrence. However, these associations may also be influenced by baseline TG levels, diabetes, obesity, diagnostic delay, and differences in treatment strategies.

Clinically, HTG‐AP often has characteristic features. These features include lipemic plasma, marked TG elevation, a background of metabolic syndrome or diabetes, obesity, and a high risk of recurrence [243, 244]. Because lipemia can interfere with laboratory assays, serum amylase levels may be only mildly elevated or lower than expected in some patients with HTG‐AP. Therefore, when AP is suspected but amylase elevation is atypical, clinicians should make an integrated assessment based on the presence of lipemia, serum lipase levels, TG levels, and imaging findings. Recurrent HTG‐AP is an important challenge in clinical management [245]. Poor long‐term TG control, uncorrected secondary triggers, poor glycemic control, persistent alcohol use, obesity, and poor adherence to long‐term lipid‐lowering therapy can all increase the risk of recurrence [246, 247, 248]. Repeated episodes can worsen local pancreatic injury and may lead to chronic pancreatitis, pancreatic exocrine insufficiency, and pancreatogenic diabetes [249, 250, 251].

The clinical significance of HTG‐AP lies not only in its acute severity but also in its tendency to recur and its potential long‐term pancreatic sequelae. Viewing HTG‐AP as an organ‐specific endpoint of severe‐to‐extreme HTG, particularly when chylomicronemia is present or persistent, helps distinguish a pancreatitis‐predominant phenotype, driven mainly by chylomicronemia and pancreatic injury, from an ASCVD‐predominant phenotype, driven mainly by the burden of atherogenic apoB‐containing particles and residual ASCVD risk.

### 4.3 Metabolic and Systemic Complications

Beyond ASCVD and AP, HTG is closely associated with systemic metabolic dysfunction. HTG is a common lipid phenotype in insulin resistance, metabolic syndrome, obesity, type 2 diabetes, and MASLD. Elevated circulating FFAs can impair insulin signaling in skeletal muscle and the liver, reduce glucose uptake, increase hepatic gluconeogenesis, and promote insulin resistance and glycemic dysregulation [252, 253]. In turn, insulin resistance enhances adipose tissue lipolysis, increases FFA delivery to the liver, and promotes hepatic TG synthesis and VLDL overproduction [254]. This process creates a vicious cycle of FFA excess, hepatic TG accumulation, increased VLDL secretion, and worsening HTG, thereby further amplifying the systemic metabolic burden [255, 256].

HTG is closely linked to MASLD. MASLD is characterized by excessive lipid accumulation in hepatocytes and shares several core mechanisms with HTG. These mechanisms include insulin resistance, increased DNL, increased VLDL production, mitochondrial dysfunction, oxidative stress, ER stress, and chronic low‐grade inflammation. These processes may drive disease progression from simple steatosis to metabolic dysfunction‐associated steatohepatitis and liver fibrosis [257, 258]. Mitochondrial injury and autophagy imbalance may also contribute to disease progression [259, 260]. The causal direction between HTG and MASLD progression is complex and may vary according to population characteristics, genetic background, and metabolic status. Therefore, in the context of MASLD, HTG should be understood as part of a broader metabolic dysfunction phenotype rather than as an isolated causal driver.

HTG also commonly coexists with CKD and other systemic metabolic disorders. Some studies suggest that TRL remnant particles may contribute to kidney injury through glomerular or renal vascular lipid deposition, mesangial cell activation, oxidative stress, inflammation, endothelial dysfunction, and fibrotic remodeling [79, 261]. HTG, MASLD, and CKD may share several mechanisms, including insulin resistance, chronic inflammation, lipotoxicity, and endothelial dysfunction [15, 262]. Compared with ASCVD and HTG‐AP, the causal evidence linking HTG to CKD progression remains insufficient. Whether TG‐targeted interventions can improve renal outcomes requires further investigation [263].

Recent observational and genetic evidence also suggests that elevated TG levels and increased RC, the latter reflecting the cholesterol burden of TRL remnants, are associated with higher risks of nontraditional outcomes such as calcific aortic valve stenosis and abdominal aortic aneurysm [264, 265]. Potential mechanisms may involve tissue lipid retention, chronic inflammation, oxidative stress, extracellular matrix remodeling, and structural changes in the vascular wall [266]. However, these associations are still based mainly on observational or genetic evidence and cannot directly establish that TG‐lowering therapy improves these clinical outcomes.

In the clinical management of HTG, pregnancy is a special stage that requires close attention. Increased estrogen and placental hormone levels during pregnancy can promote hepatic VLDL production and lead to a physiological rise in TG levels. In women with FCS, MCS, a history of severe or extreme HTG, diabetes, or obesity, pregnancy can trigger severe chylomicronemia and HTG‐AP [267]. HTG during pregnancy may also be associated with adverse pregnancy outcomes, including preeclampsia, gestational diabetes, and excessive fetal growth [268, 269]. These associations are often confounded by obesity, insulin resistance, diabetes, and other maternal risk factors. Therefore, the causal role of HTG in adverse pregnancy outcomes and the benefits of specific TG‐lowering interventions still require further validation [270, 271].

### 4.4 Phenotype‐Specific Risk Trajectories and Clinical Implications

The clinical consequences of HTG can be summarized as two major risk trajectories (Figure 2). The first is the pancreatitis‐predominant trajectory. It is characterized by severe TG elevation, chylomicronemia, accumulation of CMs and large TRLs, pancreatic microcirculatory impairment, local FFA‐mediated lipotoxicity, and amplification of acute inflammatory cascades. This trajectory mainly leads to HTG‐AP, recurrent pancreatitis, SIRS, organ dysfunction, and long‐term impairment of pancreatic endocrine and exocrine function. The second is the ASCVD‐predominant trajectory. It is characterized by an increased burden of VLDL and remnant lipoproteins, including IDL, accompanied by elevated RC and apoB‐containing particles. This trajectory promotes ASCVD risk through arterial intimal retention, cholesterol deposition, endothelial dysfunction, foam cell formation, chronic vascular inflammation, and plaque progression.

**FIGURE 2:**
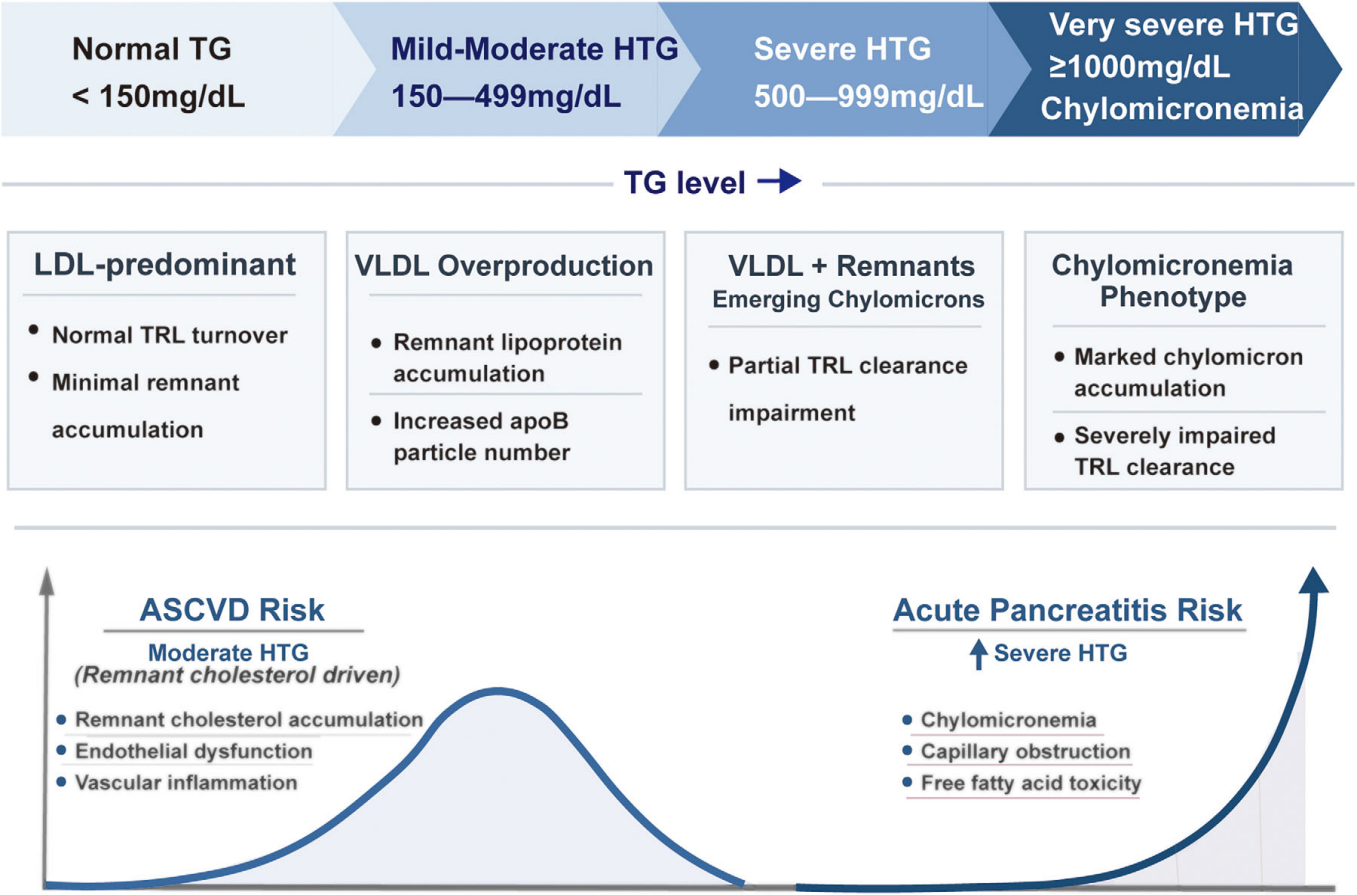
Clinical consequences across the triglyceride continuum. Increasing TG levels are accompanied by progressive changes in lipoprotein phenotype, from normal TRL turnover to VLDL overproduction, remnant accumulation, impaired TRL clearance, and chylomicronemia. Mild‐to‐moderate HTG is mainly associated with residual ASCVD risk driven by remnant cholesterol accumulation, endothelial dysfunction, and vascular inflammation, whereas severe and extreme HTG progressively increase AP risk through chylomicronemia, capillary obstruction, and FFA toxicity. This continuum highlights that TG thresholds reflect distinct metabolic phenotypes and complication profiles rather than a single uniform risk state.

Although these two trajectories share several stress and inflammatory responses, their dominant mechanisms differ. In HTG‐AP, FFA‐mediated lipotoxicity, mitochondrial dysfunction, ER stress, disturbed Ca^2^^+^ homeostasis, and activation of inflammatory signaling contribute to premature zymogen activation, acinar cell injury, necrosis, and systemic inflammatory responses [122]. In ASCVD‐predominant HTG, the central mechanisms are remnant particle retention, apoB particle accumulation, cholesterol deposition, endothelial dysfunction, foam cell formation, chronic vascular inflammation, and progression of atherosclerotic plaques [8, 213].

This framework has direct clinical translational relevance. For pancreatitis‐predominant HTG, risk assessment should emphasize peak TG levels, TG variability, chylomicronemia, a previous history of AP, genetic background, pregnancy status, uncontrolled diabetes, alcohol use, obesity, and exposure to TG‐raising drugs. The main management goal is to prevent first and recurrent episodes of AP by reducing CM burden, correcting secondary factors, and maintaining stable long‐term TG control.

For ASCVD‐predominant HTG, risk assessment centers on apoB, non‐HDL‐C, RC, LDL‐C, diabetes, CKD, metabolic syndrome, subclinical atherosclerosis, and overall ASCVD risk. Treatment goals extend beyond TG lowering to include reduction of atherogenic apoB‐containing lipoprotein particle burden and long‐term residual cardiovascular risk.

HTG management cannot be driven by TG concentration alone. Future risk stratification and treatment decisions need to integrate TG levels, lipoprotein phenotype, apoB‐containing particle burden, RC, genetic susceptibility, secondary triggers, and previous clinical events. Based on this phenotype‐oriented framework, HTG management may move from a single biochemical marker‐driven approach toward precision management centered on etiology, lipoprotein composition, and organ‐specific risk.

## 5 Diagnosis and Risk Stratification of HTG

The diagnosis and risk stratification of HTG require more than confirmation of elevated TG levels. Clinical evaluation should characterize the etiologic background, lipoprotein phenotype, secondary triggers, and predominant clinical risks. Persistent mild‐to‐moderate HTG usually reflects an increased burden of apoB‐containing TRLs and their remnants, highlighting residual ASCVD risk. Severe HTG and, particularly, extreme HTG accompanied by chylomicronemia warrant focused assessment of AP risk, potential genetic causes, and reversible secondary triggers.

Accordingly, the diagnostic pathway for HTG should emphasize structured phenotypic assessment rather than reliance on a single TG value. This section focuses on fasting and nonfasting TG measurement, diagnostic thresholds, screening for secondary causes, ASCVD and AP risk stratification, genetic testing, and prognostic assessment of HTG‐AP. It proposes a diagnostic framework that integrates TG levels, lipoprotein composition, apoB‐ containing particle burden, underlying etiology, and organ‐specific risk to support subsequent individualized management. As a practical entry point for this framework, Table 3 summarizes the clinical classification of fasting TG levels according to predominant lipoproteins and major clinical implications.

**TABLE 3:** Clinical classification of fasting TG levels by predominant lipoproteins and major clinical implications.

Triglyceride level, mg/dL (mmol/L)	Category	Predominant lipoproteins	Main clinical implications
<150 (<1.7)	Normal	Physiological VLDL predominates in the fasting state; CMs are generally absent after an adequate fasting period.	Reference range [40]
150–199 (1.7–2.2)	Mild HTG	Predominantly VLDL	Early metabolic dyslipidemia; commonly associated with insulin resistance and metabolic syndrome [40]
200–499 (2.3–5.6)	Moderate HTG	VLDL and TRL remnants	Associated with increased ASCVD risk and a greater burden of remnant lipoproteins and apoB‐containing particles [8, 12]
500–999 (5.6–11.3)	Severe HTG	Predominantly VLDL and TRL remnants; CMs may become detectable as TG levels increase, particularly in susceptible individuals.	Increasing risk of AP; secondary causes should be evaluated, and TG‐lowering strategies for pancreatitis prevention may need to be considered [12].
1000–1999 (11.3–22.6)	Chylomicronemia‐range HTG (extreme HTG)	CMs with concomitant VLDL accumulation	Substantial circulating CM accumulation is generally present; high risk of AP [5, 40].
≥2000 (≥22.6)	Chylomicronemia‐range HTG (extreme HTG), very high level	Marked accumulation of CMs, usually accompanied by VLDL	Very high risk of AP and a greater likelihood of clinical manifestations associated with chylomicronemia; the TG level alone does not establish a specific chylomicronemia syndrome [5, 40].

*Note*: This table presents the operational classification adopted in this review to ensure consistent terminology throughout the manuscript. The thresholds for mild and moderate HTG are informed by the NCEP ATP III and Endocrine Society classifications. The 2021 ACC Expert Consensus Decision Pathway broadly defines severe HTG as TG levels ≥500 mg/dL, reflecting a clinically actionable threshold. For greater terminological precision, this review further classifies TG levels of 500–999 mg/dL as severe HTG and levels ≥1000 mg/dL as extreme HTG. The latter generally corresponds to the chylomicronemia range because substantial circulating CM accumulation is usually present at this level.

The ranges of 1000–1999 mg/dL and ≥2000 mg/dL are presented separately to illustrate increasing CM accumulation, biochemical severity, and pancreatitis risk; both remain within the category of extreme HTG. A TG level ≥2000 mg/dL represents a particularly marked biochemical phenotype but does not by itself, establish FCS, MCS, or another specific chylomicronemia disorder. When specific guidelines are discussed in the text, their original terminology and TG thresholds are explicitly stated. The predominant lipoprotein patterns shown in the table are generalized descriptions and may vary according to fasting status, genetic background, insulin resistance, glycemic control, alcohol intake, medications, and other secondary factors.

Abbreviations: AP, acute pancreatitis; apoB, apolipoprotein B; ASCVD, atherosclerotic cardiovascular disease; CM, chylomicron; HTG, hypertriglyceridemia; TG, triglycerides; TRL, triglyceride‐rich lipoprotein; VLDL, very‐low‐density lipoprotein.

### 5.1 Fasting and Nonfasting TG Measurement

The clinical paradigm for TG measurement is changing, and nonfasting lipid testing is increasingly used in routine clinical practice. Historically, fasting TG measurement was considered the standard approach for the diagnosis, treatment monitoring, and phenotypic assessment of HTG. Growing evidence suggests that, in common clinical settings, nonfasting TG measurement also has diagnostic value and provides useful information for ASCVD risk assessment [272, 273, 274].

This shift has a clear physiological basis. Individuals spend much of the day in a postprandial state. Therefore, nonfasting TG levels may more closely reflect the cumulative daily exposure to TRLs and their remnants [275]. Multiple large population‐based cohort studies have shown that elevated nonfasting TG levels are associated with increased risks of coronary heart disease, ischemic stroke, cardiovascular mortality, and all‐cause mortality [276, 277]. These findings support the role of TRL remnants and RC in the development and progression of atherosclerosis. They also suggest that nonfasting TG can serve as a useful screening marker for identifying ASCVD‐predominant HTG.

Based on this evidence, several expert consensus statements and guidelines recommend that nonfasting lipid testing can be used for general cardiovascular risk assessment [28, 278]. In most outpatient screening, initial risk assessment, and long‐term ASCVD risk management settings, nonfasting TG measurement is convenient and clinically interpretable [279]. Fasting TG measurement remains important in several key clinical settings. Standardized fasting TG measurement remains necessary in several key settings. When nonfasting TG is markedly elevated, for example >5.6 mmol/L (500 mg/dL), fasting testing helps confirm the degree of elevation and reduce biological variability. When TG levels approach or exceed 11.3 mmol/L (1000 mg/dL), fasting testing is particularly important for evaluating chylomicronemia and pancreatitis risk [280, 281]. In addition, fasting TG measurement provides better comparability when clinicians systematically evaluate secondary causes, distinguish FCS from MCS, monitor the response to TG‐lowering therapy, and assess the risk of recurrent HTG‐AP [282].

Therefore, fasting and nonfasting TG measurement should be viewed as complementary tools rather than substitutes for each other. Nonfasting testing is generally suitable for routine lipid screening and ASCVD risk assessment, whereas fasting testing is preferred for confirming severe or extreme HTG, evaluating chylomicronemia, stratifying pancreatitis risk, characterizing suspected inherited HTG phenotypes, and monitoring treatment response. In patients with suspected HTG‐AP, TG levels should be measured as early as possible because fasting and treatment may rapidly lower the circulating TG concentration. When extreme HTG is suspected or the result will directly influence clinical decision‐making, standardized fasting lipid measurements should subsequently be obtained and repeated when clinically appropriate.

### 5.2 Diagnostic Thresholds and Clinical Definitions

The diagnosis of HTG is mainly based on TG levels. According to current clinical practice and major guidelines, fasting TG levels ≥1.7 mmol/L (150 mg/dL) are generally used to define HTG. HTG can then be further stratified by severity. TG levels of 1.7–5.6 mmol/L (150–499 mg/dL) usually correspond to mild‐to‐moderate HTG. TG levels ≥5.6 mmol/L (500 mg/dL) indicate more pronounced HTG and are associated with a progressively increasing risk of pancreatitis. TG levels ≥11.3 mmol/L (1000 mg/dL) usually indicate extreme HTG, are often accompanied by chylomicronemia, and require focused assessment of AP risk [26, 40].

These TG thresholds should be viewed as entry points for clinical risk assessment rather than rigid diagnostic boundaries. Patients with the same TG level may differ in lipoprotein composition, apoB particle burden, RC levels, genetic background, and secondary triggers. Their ASCVD risk and AP risk may therefore differ substantially. Stratification based only on TG values may underestimate the phenotypic heterogeneity of HTG. A comprehensive assessment should also consider the lipoprotein profile, apoB‐related markers, and clinical background [283].

The timing and clinical context of TG measurement are also important. In patients with suspected HTG‐AP, fasting and early interventions can markedly reduce TG levels within a short period. This reduction may obscure the etiologic role of HTG. In patients with acute abdominal pain, suspected AP, or lipemic plasma, early lipid testing can facilitate etiologic diagnosis [284]. During follow‐up and treatment monitoring, testing should be performed under relatively consistent conditions whenever possible. For example, maintaining consistent fasting or nonfasting status can improve the comparability of results across time points [281].

Diagnostic evaluation should also include screening for secondary causes. Uncontrolled diabetes, obesity, metabolic syndrome, hypothyroidism, CKD, MASLD, excessive alcohol use, pregnancy, and TG‐raising drugs can all trigger or worsen HTG [51, 285]. Identifying these factors is important for assessing the reversibility of HTG, the risk of recurrence, and treatment priorities.

### 5.3 Etiologic Assessment and Risk Phenotyping

The goal of diagnostic evaluation in HTG is not only to confirm TG elevation. It should also address three interrelated clinical questions: whether TG elevation is persistent and how severe it is; whether the main drivers are genetic, metabolic, drug‐related, physiological, or multifactorial; and whether the patient's current predominant clinical risk is ASCVD, AP, or both.

ASCVD risk should be assessed systematically, especially in patients with mild‐to‐moderate or persistent HTG. These patients often have increased levels of VLDL and TRL remnants, together with elevated RC. They may also have an increased burden of apoB‐containing particles. Assessment based on LDL‐C alone may underestimate the total atherogenic lipoprotein burden. Non‐HDL‐C and apoB better reflect the total burden of all apoB‐containing lipoprotein particles, especially in patients with elevated TG, metabolic syndrome, diabetes, or mixed dyslipidemia. RC, as a marker of the cholesterol burden carried by TRL remnants, is also becoming an important complementary measure in the assessment of residual ASCVD risk [286, 287].

When interpreting LDL‐C in patients with HTG, clinicians should recognize the limitations of estimation methods. The traditional Friedewald formula relies on TG/5 (mg/dL) to estimate very‐low‐density lipoprotein cholesterol (VLDL‐C). Its accuracy decreases when TG levels are elevated, in the postprandial state, or when VLDL composition is abnormal. This limitation is especially important when TG levels are markedly elevated, because LDL‐C may be underestimated [288, 289]. In patients with elevated TG, diabetes, metabolic syndrome, or mixed dyslipidemia, relying only on estimated LDL‐C may underestimate the burden of apoB‐containing atherogenic particles [290]. Therefore, in the risk assessment of ASCVD‐predominant HTG, non‐HDL‐C, apoB, and RC can provide more comprehensive risk information than estimated LDL‐C alone [291]. When TG levels are markedly elevated or when treatment decisions are influenced by LDL‐C, clinicians may consider direct LDL‐C measurement or LDL‐C estimation methods that are better suited to the context of HTG, such as the Martin/Hopkins or Sampson/NIH equations [292].

For ASCVD‐predominant HTG, risk assessment should integrate TG, LDL‐C, non‐HDL‐C, apoB, RC, diabetes status, blood pressure, smoking status, kidney function, and history of ASCVD. This assessment framework helps avoid underestimation of residual cardiovascular risk based only on TG levels or estimated LDL‐C. It also more accurately reflects the burden of apoB‐containing particles.

AP risk assessment is warranted when TG levels are markedly elevated, approach or exceed 11.3 mmol/L (1000 mg/dL), or when symptoms suggestive of pancreatitis, such as abdominal pain, nausea, or vomiting, are present. The assessment of pancreatitis‐predominant HTG focuses on peak TG levels, TG variability, the presence and persistence of chylomicronemia, lipemic plasma, previous AP, the possibility of FCS or MCS, and secondary factors capable of triggering a rapid increase in TG levels. Clinical manifestations associated with chylomicronemia may include recurrent abdominal pain, AP, eruptive xanthomas, lipemia retinalis, and hepatosplenomegaly. In patients with suspected AP, lipase, amylase, liver function tests, serum calcium, and blood glucose should be promptly assessed. These results are interpreted together with imaging findings to support an integrated clinical assessment [234].

Secondary factors are very common in HTG, and many of them are modifiable. The evaluation should focus on glycemic control, obesity and metabolic syndrome, hypothyroidism, CKD, MASLD, excessive alcohol use, pregnancy status, and dietary factors. Medication history is also critical. Relevant drugs include estrogen preparations, glucocorticoids, retinoids, atypical antipsychotic drugs, some β‐blockers, thiazide diuretics, immunosuppressive agents, and antiviral drugs [51]. In patients with marked TG elevation or a recent rapid increase in TG levels, identifying and correcting these triggers often has direct clinical value.

Inherited lipid disorders should be considered in patients with early‐onset, severe, persistent, or refractory HTG. This is especially important in patients with recurrent pancreatitis, chylomicronemia, onset in childhood or young adulthood, extremely high TG levels at a low body mass index, familial clustering of dyslipidemia, or premature ASCVD [43]. In addition to TG measurement, the basic laboratory evaluation should usually include a complete lipid profile, apoB or non‐HDL‐C, liver and kidney function tests, thyroid‐stimulating hormone, glycated hemoglobin, fasting glucose, urine protein or albuminuria assessment, and pregnancy testing when appropriate [293].

This structured diagnostic pathway helps connect TG elevation with etiology, lipoprotein phenotype, and organ‐specific risk, thereby providing a basis for individualized treatment.

### 5.4 Clinical Role of Genetic Testing in HTG

Genetic testing is increasingly used in the diagnosis and management of selected HTG phenotypes. Its clearest clinical value is in the evaluation of patients with suspected FCS or PC [294]. FCS is a monogenic form of chylomicronemia with a relatively well‐defined molecular basis. It is usually caused by biallelic pathogenic variants in genes related to the LPL pathway. Pathogenic variants in LPL are the most common, and other related genes include APOC2, APOA5, GPIHBP1, and LMF1 [295].

Genetic testing has several important clinical implications. Identification of biallelic pathogenic variants affecting the LPL pathway can support the diagnosis of FCS and help distinguish it from MCS and other multifactorial forms of HTG that may present with overlapping biochemical and clinical features [296]. FCS typically presents with persistent extreme HTG and fasting chylomicronemia, often beginning in childhood or early adulthood, together with recurrent abdominal pain or pancreatitis, a limited response to conventional TG‐lowering therapies, and apoB levels that are relatively low in relation to the marked TG elevation. By contrast, MCS more commonly occurs in individuals with polygenic susceptibility combined with secondary or metabolic factors, such as obesity, diabetes, alcohol consumption, pregnancy, or medication exposure. TG levels are generally more variable in MCS, apoB levels tend to be higher because of concomitant VLDL and remnant particle accumulation, and other cardiometabolic risk factors are frequently present. An integrated assessment of apoB, the relationship between TG and apoB, the persistence and variability of TG elevation, age at onset, secondary factors, treatment response, clinical history, and genetic findings can help differentiate FCS from MCS and other multifactorial forms of HTG.

Genetic diagnosis can support familial risk identification, genetic counseling, long‐term dietary management, and treatment decision‐making. Because the core defect in FCS is severe impairment of the LPL pathway, patients with FCS often have limited responses to conventional TG‐lowering therapies, particularly therapies whose effects depend substantially on residual LPL activity, such as fibrates. In recent years, mechanism‐guided therapies such as apoC‐III inhibition have provided new treatment options for patients with FCS and some patients with severe HTG [297, 298]. Genetic diagnosis may therefore influence patient selection, treatment expectations, and long‐term management strategies.

Genetic testing should be considered in patients with extreme HTG or PC, particularly when accompanied by recurrent AP of otherwise unclear etiology, onset in childhood or early adulthood, familial clustering of chylomicronemia or HTG‐AP, limited response to conventional TG‐lowering therapy, or other clinical features suggestive of FCS [299]. Most cases of mild‐to‐moderate HTG result from the combined effects of polygenic and environmental factors. These patients usually do not require routine genetic testing, because genetic testing has limited immediate impact on daily clinical management in this setting. In the future, polygenic risk scores may help quantify genetic susceptibility and improve risk prediction, but they currently remain mainly in the research or exploratory stage [67, 300].

Targeted gene panels based on next‐generation sequencing have become an important strategy for the molecular diagnosis of inherited lipid disorders [301]. Interpretation of results can be complex, and variants of uncertain significance are not uncommon. Genetic testing results should be interpreted together with TG levels, apoB, lipoprotein phenotype, age at onset, history of pancreatitis, secondary factors, and family history. This integrated approach can help avoid overdiagnosis or treatment decisions based only on a single genetic variant [302].

### 5.5 Severity and Prognostic Assessment in HTG‐AP

In patients with confirmed or strongly suspected HTG‐AP, clinical evaluation should not only establish HTG as the etiology but also include severity stratification and prognostic assessment [303, 304]. TG levels and chylomicronemia status can help determine the etiology, but they do not accurately predict AP severity. Severity and prognostic assessment in HTG‐AP should integrate clinical presentation, organ function, inflammatory response, imaging findings, and metabolic status.

Among biochemical markers, serum lipase generally has greater diagnostic sensitivity and specificity than amylase and is the preferred pancreatic enzyme test in patients with suspected AP [305, 306, 307]. However, lipase elevation alone is not sufficient to establish the diagnosis and should be interpreted together with the clinical presentation and, when necessary, imaging findings. In extreme HTG with marked lipemia, analytical interference may produce inaccurate results in several laboratory assays, including measurements of amylase, lipase, sodium, bilirubin, and other biochemical analytes. Therefore, normal or only mildly elevated pancreatic enzyme levels do not exclude HTG‐AP. In this setting, laboratory results should be interpreted in conjunction with the timing of sample collection, the degree of lipemia, clinical findings, and pancreatic imaging [308, 309, 310]. C‐reactive protein and procalcitonin are not specific markers of HTG‐AP. However, they can serve as adjunctive markers for assessing the degree of systemic inflammation, infectious complications, and the risk of poor prognosis [311, 312].

Clinical scoring systems are important tools for assessing the severity of AP. The Bedside Index for Severity in Acute Pancreatitis (BISAP), Acute Physiology and Chronic Health Evaluation II (APACHE II), and Ranson score are all used for clinical risk stratification [313]. BISAP is suitable for rapid assessment early after admission. APACHE II is more complex, but it can dynamically reflect the overall physiological status of critically ill patients. The Ranson score requires data collected over 48 h, which limits its convenience for early decision‐making [314].

Most of these scoring systems were developed in general AP populations and do not fully incorporate metabolic abnormalities and lipid‐related parameters that are especially relevant to HTG‐AP. These include peak TG levels, chylomicronemia, diabetic ketoacidosis, severe insulin resistance, and lipemia‐related assay interference. Their predictive performance in HTG‐AP may therefore be inconsistent [315]. This limitation suggests the need to develop or validate risk assessment tools that are better suited to the pathophysiological background of HTG‐AP. Such tools should combine traditional AP severity indicators with dynamic changes in TG levels, metabolic control status, and lipoprotein phenotype.

Artificial intelligence (AI) and machine learning are increasingly being investigated for risk prediction in HTG, and for severity and prognostic assesment in HTG‐AP. Existing models can be broadly divided into models based on clinical and laboratory variables and models that integrate imaging features, dynamic disease‐course data, and multimodal information. These approaches may capture nonlinear relationships that are difficult to identify using traditional scoring systems. In some studies, they have shown good performance in severity prediction and prognostic stratification [316, 317, 318, 319, 320]. At present, most AI models are still based on single‐center retrospective datasets. External validation remains insufficient, and model interpretability, standardization of input variables, and real‐world feasibility are still limited [321, 322]. Therefore, AI should be regarded as an auxiliary and research‐oriented tool rather than a standard method that can replace clinical judgment or established scoring systems. Future studies should rely on large, multicenter, standardized datasets. Priority should be given to the development of interpretable and externally validated prediction models that can be embedded into clinical workflows [323].

### 5.6 Integrated Diagnostic Framework and Clinical Implications

The diagnosis and risk stratification of HTG need to move beyond assessment based on a single TG value toward multidimensional phenotypic assessment. A practical diagnostic framework first confirms the persistence and severity of TG elevation, and then integrates testing conditions, lipoprotein composition, apoB‐containing particle burden, RC, secondary factors, genetic background, and previous clinical events. This approach can help determine whether the patient is mainly on an ASCVD‐predominant trajectory, a pancreatitis‐predominant trajectory, or both.

This integrated diagnostic perspective has direct clinical relevance. For patients whose main risk is ASCVD, diagnostic evaluation supports long‐term cardiovascular risk management. For patients whose main risk is chylomicronemia and AP, diagnostic evaluation supports trigger identification, genetic classification, acute risk assessment, and recurrence prevention. Therefore, the diagnosis of HTG should extend beyond determining whether TG levels are elevated. It also needs to answer why TG levels are elevated, which lipoprotein abnormalities are driving the phenotype, which organ‐specific risk predominates, and which management strategy deserves priority. Linking upstream causes, the current lipoprotein phenotype, organ‐specific risk, and subsequent treatment decisions can help avoid oversimplified management based only on TG levels. It may also support more precise prevention of HTG‐AP, reduction of ASCVD risk, and improvement of long‐term metabolic outcomes.

## 6 Management Strategies for HTG

The management of HTG requires a shift from a single TG threshold‐driven approach to stratified management centered on the dominant clinical risk, lipoprotein phenotype, etiologic background, and treatment goals [8, 12, 13]. Lifestyle intervention, weight management, alcohol abstinence, and correction of secondary factors form the foundation of treatment across HTG phenotypes. Pharmacological therapy and emerging targeted therapies should be individualized according to ASCVD risk, pancreatitis risk, genetic background, and treatment availability [324].

Treatment of HTG is best conceptualized not as simple “normalization” of TG levels, but as risk‐oriented intervention. In ASCVD‐predominant HTG, therapy focuses on reducing apoB‐containing atherogenic lipoprotein burden, as reflected by non‐HDL‐C and apo B, thereby lowering long‐term ASCVD risk. In pancreatitis‐predominant HTG, therapy prioritizes lowering peak TG levels, reducing CM burden, correcting secondary triggers, and preventing first or recurrent episodes of HTG‐AP.

### 6.1 Risk‐Stratified Management Framework

The management of HTG begins with defining the patient's current dominant risk phenotype. Treatment decisions can be guided by three core questions: whether immediate pancreatitis risk requires urgent intervention; whether long‐term ASCVD risk warrants sustained risk reduction; and which genetic, metabolic, drug‐related, or lifestyle factors are driving TG elevation. For patients with ASCVD‐predominant risk, lipid management is best incorporated into a comprehensive cardiovascular risk intervention framework [325, 326]. For patients with pancreatitis‐predominant risk, especially those with severe or extreme HTG or a history of HTG‐AP, the priority is to lower peak TG levels and reduce or prevent chylomicronemia [87].

For patients with established HTG‐AP, management is further staged according to the disease course. During the acute phase, care relies on standard supportive care for AP and metabolic stabilization, with TG‐lowering measures used when clinically appropriate. During remission, management focuses on preventing TG rebound, controlling secondary triggers, identifying inherited causes, and reducing the risk of long‐term recurrence [87].

Overall, management goals need to align with the dominant risk phenotype rather than TG level alone. The selection of conventional pharmacological and emerging targeted therapies depends on the relevant clinical objective and pathophysiological context [327, 328].

### 6.2 Acute Management of Extreme HTG and HTG‐AP

Acute‐phase management mainly applies to patients requiring urgent evaluation or hospitalization because of extreme HTG, marked chylomicronemia, or confirmed or strongly suspected HTG‐AP. The goals are to manage AP‐related systemic inflammatory responses and stabilize organ function, correct metabolic factors that trigger a rapid rise in TG levels, and, when appropriate, reduce circulating TG levels and TRL burden. Although rapid TG reduction has clear biological plausibility, the decrease in TG levels itself should not be directly equated with improvements in mortality, organ failure, intensive care unit (ICU) length of stay, or long‐term complications. Acute TG‐lowering interventions should be considered part of comprehensive supportive care for AP rather than a substitute for standard AP treatment. For HTG‐AP, the core elements of acute management remain early severity assessment, maintenance of tissue perfusion, pain control, nutritional support, monitoring of organ function, and correction of triggering factors.

#### 6.2.1 Supportive Care Remains the Cornerstone of Acute Management

HTG‐AP remains part of the broader AP spectrum. Therefore, acute‐phase treatment should first follow the principles of supportive care for AP, including early risk assessment, fluid resuscitation, adequate analgesia, nutritional support, organ function monitoring, and management of complications [237]. Fluid resuscitation helps maintain pancreatic microcirculation and systemic tissue perfusion [329]. Lactated Ringer's solution is often considered a reasonable choice for initial fluid resuscitation in AP [330, 331]. Goal‐directed fluid therapy should be adjusted dynamically according to hemodynamic status, urine output, hematocrit, blood urea nitrogen, and cardiac and kidney function. This approach helps avoid both under‐resuscitation and fluid overload [332].

Early enteral nutrition is preferred over parenteral nutrition. It helps maintain intestinal mucosal barrier integrity and reduces the risk of infectious complications. In patients with mild disease who can resume oral intake, a low‐fat diet can be gradually reintroduced. In patients who cannot tolerate oral feeding, nasogastric or nasojejunal enteral nutrition should be prioritized. Parenteral nutrition should be considered only when enteral nutrition is not tolerated or is clearly contraindicated [333].

Pain management is an important component of acute‐phase treatment for AP. Persistent severe abdominal pain not only reduces quality of life [334] but may also contribute to disease activity through stress responses and neurogenic inflammation [335, 336]. Opioids are commonly used for pain control in moderate‐to‐severe AP, but clinicians should be aware of the risks of respiratory depression, reduced intestinal motility, and dependence. Nonsteroidal anti‐inflammatory drugs may be an option for mild‐to‐moderate pain. Some studies suggest that their analgesic efficacy may be comparable to that of opioids, with potential additional anti‐inflammatory effects [337, 338, 339]. Epidural analgesia has also been used to reduce opioid requirements and may improve visceral perfusion in selected patients [340, 341, 342]. The analgesic regimen can be individualized according to pain intensity, organ function, bleeding risk, and local clinical availability.

HTG‐AP is usually a sterile inflammatory process. Therefore, routine prophylactic antibiotic use is not recommended [237, 343]. Antibiotic therapy is reserved for patients with documented extrapancreatic infection or a high suspicion of infectious complications, such as infected necrosis, biliary infection, or sepsis [344, 345].

#### 6.2.2 Insulin Therapy in Acute Management

Insulin is frequently used as a metabolic intervention in selected hospitalized patients with extreme HTG or HTG‐AP, particularly in the presence of diabetes, marked hyperglycemia, or diabetic ketoacidosis [244, 346, 347, 348]. Insulin can lower TG levels by enhancing LPL activity, suppressing adipose tissue lipolysis, reducing FFA delivery to the liver, and promoting TRL lipolysis and clearance [349, 350]. Continuous intravenous insulin infusion can markedly reduce TG levels within 24–72 hours [351].

Unlike extracorporeal removal methods such as therapeutic plasma exchange (TPE), insulin lowers TG mainly by correcting abnormal metabolic states and improving TRL clearance, rather than by directly removing circulating lipoproteins. Therefore, insulin therapy is particularly suitable for HTG‐AP driven by metabolic decompensation. Insulin therapy requires close monitoring of blood glucose, electrolytes, and fluid status, with attention to the risks of hypoglycemia and hypokalemia [352]. In patients without diabetes or with normal glucose levels, insulin requires greater caution and individualized assessment based on TG levels, disease severity, and monitoring capacity. After stabilization of the acute condition, intravenous insulin can be discontinued or transitioned to subcutaneous insulin when indicated, while management shifts toward long‐term etiologic management, lifestyle intervention, and sustained lipid‐lowering therapy [238].

#### 6.2.3 Heparin is Not Recommended as Routine TG‐Lowering Therapy

Heparin has historically been used to promote TG reduction. Its proposed mechanism is the release of endothelial surface‐bound LPL into the circulation. However, this effect is usually transient and may lead to depletion of endothelial LPL, which may impair sustained TRL clearance [353]. In addition, heparin can increase bleeding risk and should be used with particular caution in patients with AP, especially those with necrotizing pancreatitis or those who require invasive procedures. Therefore, heparin is not currently recommended as a routine TG‐lowering therapy for HTG‐AP [20].

Low‐molecular‐weight heparin (LMWH) has been hypothesized to improve outcomes in moderate‐to‐severe AP through effects on microcirculation and inflammation [354, 355]. However, a multicenter randomized clinical trial in HTG‐AP did not show that LMWH combined with insulin was superior to insulin alone. Current evidence is insufficient to support LMWH as a routine early TG‐lowering therapy for HTG‐AP [356]. If patients have venous thromboembolism, atrial fibrillation, a mechanical heart valve, or another clear indication for anticoagulation, anticoagulant therapy is guided by the primary indication, bleeding risk, and relevant specialty guidelines. Heparin or other anticoagulants should not be used as a routine strategy solely to lower TG levels.

#### 6.2.4 TPE: Marked Biochemical Efficacy but Uncertain Clinical Benefit

TPE can physically remove circulating TRLs, including CMs and VLDL, and thereby rapidly reduce TG levels. TG levels can decrease markedly after a single TPE session, with some studies reporting reductions of 50–70% [346]. In patients with very extreme HTG, marked chylomicronemia, hyperviscosity, or severe metabolic decompensation, this rapid biochemical improvement may be clinically useful for short‐term stabilization [244, 357].

The biochemical efficacy of TPE does not necessarily translate into proven clinical benefit. Existing studies have not consistently shown that TPE improves mortality, organ failure, ICU length of stay, total hospital stay, or long‐term recurrence risk. Current evidence is mainly derived from case reports, small retrospective studies, observational cohorts, and limited comparative studies. High‐quality randomized controlled trials remain limited. Therefore, TPE is not recommended as a routine first‐line treatment strategy for all patients with HTG‐AP [284, 358, 359, 360, 361, 362, 363].

A more appropriate role for TPE is as an adjunctive treatment option in highly selected patients, rather than as standard therapy. For example, TPE may be considered in HTG‐AP complicated by persistent organ failure, persistent extreme HTG despite conventional treatment, pregnancy‐associated HTG‐AP with extreme HTG or clinical deterioration, suspected hyperviscosity‐related complications, or other life‐threatening metabolic complications. The decision to use TPE requires multidisciplinary assessment of disease severity, the response to initial treatment, treatment availability, bleeding and catheter‐related risks, resource requirements, and patient preferences. More rapid TG lowering with TPE should not be assumed to translate into improved clinical outcomes.

#### 6.2.5 Early Transition to Long‐Term Management

After acute stabilization, management can transition from short‐term metabolic control to long‐term recurrence prevention as early as clinically appropriate. Before discharge, clinicians need to systematically identify uncontrolled diabetes, alcohol use, pregnancy‐related risk, drug exposure, obesity, hypothyroidism, and other secondary factors, while establishing a long‐term plan for diet, pharmacological therapy, and follow‐up. This transition from acute‐phase care to long‐term management is particularly important in patients with recurrent HTG‐AP, PC, or suspected inherited HTG [237, 245].

### 6.3 Long‐Term Management

The goals of long‐term management are to maintain sustained TG control, reduce the risk of recurrent HTG‐AP, and lower residual ASCVD risk in appropriate patients. Unlike acute‐phase treatment, long‐term management relies more on lifestyle intervention, correction of secondary factors, long‐term pharmacological therapy, and optimization of adherence.

#### 6.3.1 Lifestyle Intervention is Foundational for all HTG Phenotypes

Lifestyle intervention is the foundation of treatment for all patients with HTG. In patients with mild‐to‐moderate HTG, lifestyle modification may reduce TG levels by approximately 20–50% [364, 365]. Dietary modification needs to be individualized according to HTG severity and risk phenotype. In mild‐to‐moderate HTG, dietary intervention focuses on restricting refined carbohydrates and added sugars, especially fructose; reducing trans fats and excessive saturated fat intake; increasing dietary fiber; and replacing some saturated fat with unsaturated fats. In patients with extreme HTG, chylomicronemia, or recurrent HTG‐AP, a strict low‐fat or very‐low‐fat diet is recommended to reduce CM production and AP risk [366]. Patients with FCS usually require a long‐term very‐low‐fat diet and alcohol avoidance. For patients with extreme HTG or difficulty implementing dietary changes, involvement of a registered dietitian is particularly valuable for developing an individualized nutrition plan.

Weight management is also important. In patients with overweight or obesity, even a 5–10% reduction in body weight can produce a clinically meaningful reduction in TG levels [367]. Regular aerobic exercise can further improve insulin sensitivity and TG levels. At least 150 min of moderate‐intensity activity per week is generally recommended, with a feasible plan tailored to the patient's cardiovascular status and physical capacity [368]. Alcohol consumption can substantially aggravate TG elevation and precipitate HTG‐AP in susceptible individuals. Alcohol intake should therefore be restricted or avoided in patients with mild‐to‐moderate HTG and completely avoided in those with severe or extreme HTG, chylomicronemia, or a previous episode of HTG‐AP [369].

Lifestyle intervention also requires systematic identification and correction of secondary triggers. These triggers include poor glycemic control, obesity, hypothyroidism, CKD, MASLD, pregnancy‐related factors, and TG‐raising medications. Addressing these factors can improve the effectiveness and durability of pharmacological therapy [370].

#### 6.3.2 Statins: Cornerstone of ASCVD Risk Management

In patients with ASCVD‐predominant HTG, statins remain the foundation of therapy for reducing the risk of cardiovascular events [371]. Their main role is not simply to lower TG levels, but to reduce LDL‐C, non‐HDL‐C, and the burden of apoB‐containing lipoprotein particles [372, 373].

Although statins can reduce TG levels by approximately 10–30% by decreasing hepatic VLDL production [374], statin monotherapy is usually insufficient to achieve the degree of TG control required for pancreatitis prevention in patients with severe or extreme HTG [375]. Therefore, the role of statins in HTG management depends on the dominant risk phenotype. In pancreatitis‐predominant HTG, statins cannot replace TG‐lowering therapy directed at severe or extreme TG elevation and, when present, chylomicronemia. In patients with both ASCVD risk and severe or extreme HTG, additional TG‐lowering therapy is often needed alongside statin therapy. Regular safety monitoring is important, particularly for myopathy and liver enzyme abnormalities when statins are combined with fibrates [376].

#### 6.3.3 Fibrates: TG Lowering and Pancreatitis‐Risk Management

Fibrates lower TG levels by activating peroxisome proliferator‐activated receptor‐α. This activation enhances fatty acid oxidation, increases LPL activity, reduces apoC‐III expression, and promotes TRL clearance [377]. Fibrates usually reduce TG levels by approximately 30–50%, and therefore remain important in patients with severe or extreme HTG, especially in patients at risk of pancreatitis [378].

In pancreatitis‐predominant HTG, fibrates are commonly used for long‐term TG lowering, reduction of CM burden, and management of recurrence risk [285, 379]. They are particularly useful in patients with multifactorial extreme HTG, including those with MCS. In patients with severe defects in the LPL pathway, such as FCS, the efficacy of fibrates is usually limited because their action depends on some degree of residual LPL function. In ASCVD‐predominant HTG, the overall benefit of fibrates on ASCVD events has been inconsistent. However, fibrates may have value in selected subgroups with elevated TG and low HDL‐C [380, 381, 382].

When statins and fibrates are combined, safety should be prioritized. Fenofibrate is generally more suitable for use with statins than gemfibrozil, because gemfibrozil is associated with a higher risk of myopathy and rhabdomyolysis. In patients with impaired kidney function, fibrates should be dose‐adjusted or avoided, and creatine kinase, liver function, and kidney function should be monitored [376, 383].

#### 6.3.4 Omega‐3 Fatty Acids: Effective TG Lowering But Formulation‐Specific Clinical Value

Omega‐3 fatty acids lower TG levels by reducing hepatic TG synthesis and VLDL secretion, enhancing fatty acid oxidation, and promoting postprandial TRL clearance [384, 385]. Prescription‐grade omega‐3 fatty acids can be used for TG control in patients with severe or extreme HTG, especially when TG levels remain elevated despite lifestyle intervention and other pharmacological therapies.

The clinical implications of different omega‐3 formulations are not the same. Icosapent ethyl,a highly purified eicosapentaenoic acid (EPA) ethyl ester, has been shown to reduce ASCVD events in selected high‐risk patients, and this benefit may not be fully explained by TG reduction alone [37, 386]. In contrast, combined EPA and docosahexaenoic acid formulations can lower TG levels [387] but have not consistently shown clear cardiovascular outcome benefits [388, 389]. In ASCVD‐predominant HTG, “TG lowering” should be distinguished from “ASCVD event reduction.” In pancreatitis‐predominant HTG, prescription omega‐3 fatty acids can be used as adjunctive therapy to reduce TG burden, but their effects on HTG‐AP recurrence and hard clinical outcomes still require further evidence.

#### 6.3.5 Niacin: Limited Use in Contemporary HTG Management

The role of niacin in contemporary HTG management has become markedly limited. Although niacin can lower TG levels and increase HDL‐C, its tolerability is poor. It can cause flushing, hepatotoxicity, worsening glycemic control, and elevated uric acid levels [390, 391]. In the setting of modern statin therapy, niacin has also failed to show consistent additional ASCVD outcome benefits [392, 393]. Therefore, niacin is not routinely recommended for long‐term management of HTG or residual ASCVD risk. In selected cases, the potential use of niacin should be based on an individualized risk–benefit assessment, with close monitoring of liver function, glucose levels, and uric acid levels.

#### 6.3.6 Combination Therapy: Phenotype‐Specific Indications

Some patients may require combination therapy, especially those with both residual ASCVD risk and severe TG elevation, or those with a history of HTG‐AP [394]. In patients with high ASCVD risk and persistently elevated TG levels, high‐purity EPA may be considered in addition to statin therapy. Fibrates may also be considered in selected lipid phenotypes to reduce TG levels and TRL burden [388, 395]. In patients with extreme HTG or a history of HTG‐AP, lifestyle intervention, fibrates, omega‐3 fatty acids, optimization of diabetes treatment, and, when necessary, mechanism‐guided targeted therapies may be used to reduce peak TG levels and recurrence risk.

Combination therapy requires a clearly defined phenotype, explicit treatment goals, and careful safety assessment. Indiscriminate treatment intensification is inappropriate [396, 397]. Key considerations include drug–drug interactions, myopathy risk, liver and kidney function, glycemic changes, bleeding risk, treatment cost, and patient adherence.

#### 6.3.7 Adherence and Long‐Term Care Strategies

Long‐term patient adherence is a key determinant of success in HTG management. HTG is often a chronic metabolic disorder, and treatment efficacy depends on sustained dietary control, weight management, alcohol abstinence, adherence to pharmacological therapy, and regular follow‐up. However, these measures are often difficult to maintain in real‐world practice [293]. Difficulty adhering to dietary restrictions, insufficient physical activity, drug‐related adverse effects, treatment costs, limited awareness of disease risk, and interruption of follow‐up can all lead to TG rebound and recurrent HTG‐AP.

Therefore, long‐term management needs to explicitly address treatment adherence. Effective strategies include patient education, individualized dietary plans, feasible exercise programs, simplified medication regimens, management of adverse effects, social and family support, and multidisciplinary collaboration. In patients with a history of HTG‐AP, recurrence prevention depends on careful trigger identification and risk communication. Digital health tools, remote follow‐up, dietary records, medication reminders, and regular lipid monitoring may also improve long‐term adherence and management efficiency [398, 399].

### 6.4 Management of Recurrent HTG‐AP

Recurrent HTG‐AP is an important clinical problem in the management of extreme HTG. It can increase the burden of recurrent hospitalization and may lead to chronic pancreatitis, pancreatic exocrine insufficiency, and pancreatogenic diabetes [400, 401, 402]. Recurrence is usually associated with the combined effects of poor long‐term TG control, persistent secondary factors, and genetic susceptibility.

Established risk factors for recurrence include poor glycemic control, inadequate postdischarge TG control, persistent alcohol use, obesity, pregnancy, exposure to TG‐raising drugs, poor dietary adherence, and genetic chylomicronemia (e.g., FCS) or multifactorial chylomicronemia (e.g., MCS) [403, 404, 405, 406, 407]. Recurrence prevention cannot rely solely on rapid TG reduction during the acute phase; instead, it requires a sustained long‐term management.

TG levels are generally targeted below 5.6 mmol/L (500 mg/dL) to reduce the risk of severe TG elevation, chylomicronemia and recurrent AP. When feasible, further TG reduction and avoidance of large TG fluctuations are desirable [248, 408]. Long‐term prevention strategies include a very‐low‐fat or low‐fat diet, alcohol abstinence, optimized diabetes management, weight control, correction of secondary factors, and continued use of fibrates, prescription omega‐3 fatty acids, or other appropriate TG‐lowering therapies. In patients with genetic or refractory HTG, or recurrent HTG‐AP, referral to a lipid specialist or a specialist in genetic metabolic disorders is appropriate. Evaluation for mechanism‐guided targeted therapies may also be warranted to further reduce TG burden and recurrence risk [297, 409].

Regular follow‐up is essential for the early detection of TG rebound and metabolic imbalance [410]. Follow‐up frequency depends on TG levels, recurrence history, genetic background, treatment changes, and adherence. Lipid levels, glucose levels, and relevant secondary factors require closer monitoring early after an acute episode. Once TG levels become stable, the follow‐up interval can be gradually extended. Patients with recurrent HTG‐AP, PC, or poor treatment adherence require more frequent follow‐up, with ongoing assessment of diet, alcohol use, medication adherence, and metabolic control.

### 6.5 Emerging Targeted Therapies

With advances in the biology of TRL metabolism and human genetics, targeted therapies directed at apoC‐III, ANGPTL3, and other key regulatory factors are changing the treatment landscape for severe, inherited, or refractory HTG (Figure 3) [411]. Human genetic studies have shown that variants in genes such as APOC3, ANGPTL3, APOA5, and LPL are associated with TG levels, TRL clearance, and in some cases, ASCVD risk. These findings provide a biological rationale for targeted intervention [412, 413, 414].

**FIGURE 3:**
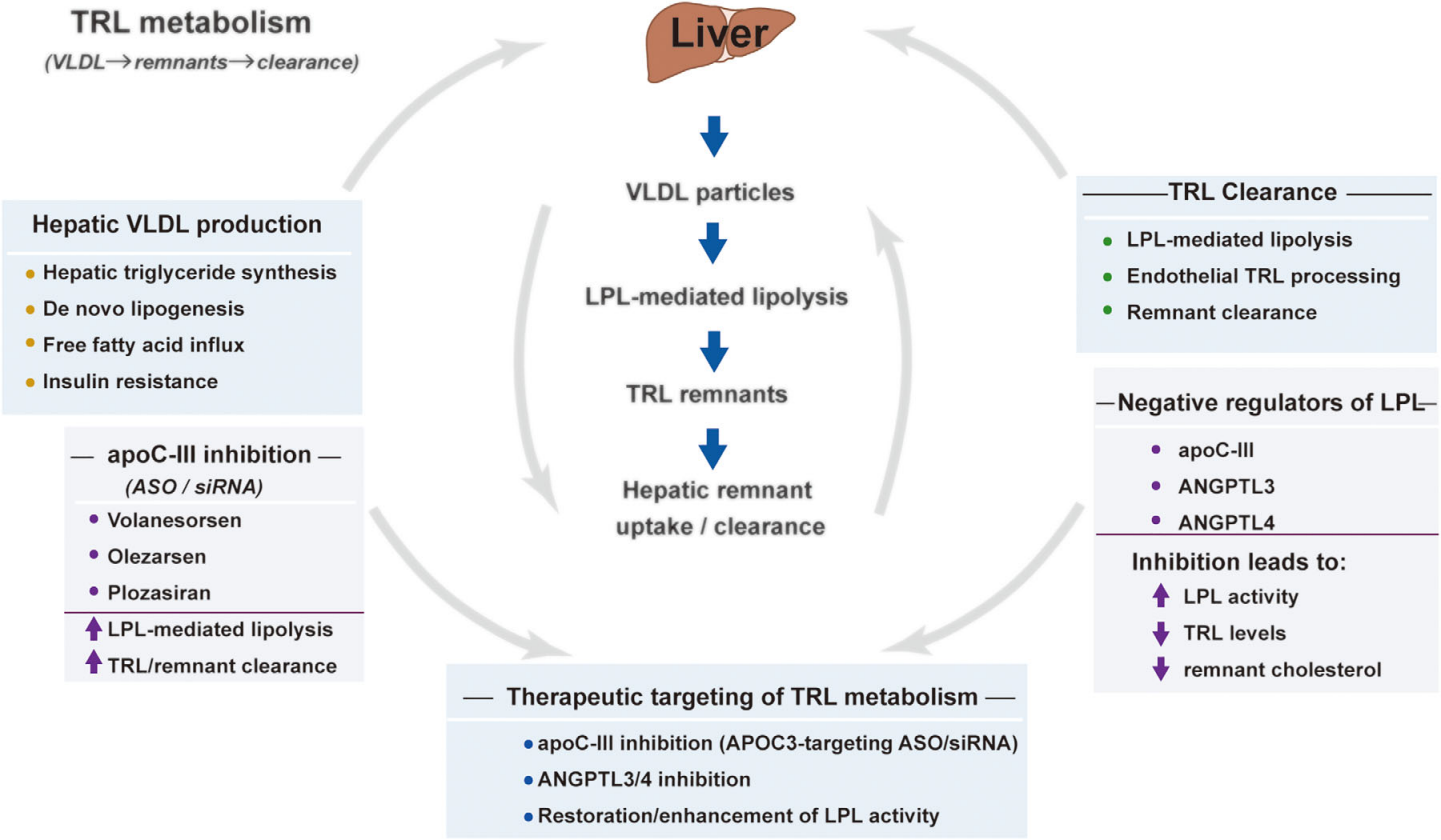
Mechanistic overview of emerging therapeutic targets in triglyceride‐rich lipoprotein metabolism. TRL metabolism involves hepatic VLDL production, LPL‐mediated lipolysis, formation of TRL remnants, and hepatic remnant uptake or clearance. Negative regulators of the LPL pathway, including apoC‐III, ANGPTL3, and ANGPTL4, impair TRL clearance and increase plasma TG levels. Emerging RNA‐based therapeutics, including antisense oligonucleotides and siRNAs targeting APOC3, ANGPTL3, and related pathways, may reduce VLDL secretion, enhance TRL clearance, and support precision lipid therapy.

A clear distinction must be made between biochemical efficacy and clinical benefit. Marked reductions in TG, apoC‐III, ANGPTL3, or TRL remnants demonstrate substantial pharmacological effects. However, they do not automatically translate into reductions in pancreatitis recurrence, ASCVD events, mortality, or organ injury. Table 4 summarizes the current status, TG‐lowering efficacy, and key limitations of major targeted therapies.

**TABLE 4:** Approved and emerging targeted therapies for hypertriglyceridemia and chylomicronemia: regulatory status, triglyceride‐lowering efficacy, clinical outcome evidence, and key limitations.

Target/pathway	Drug/therapeutic agent	Modality	Regulatory status/stage of development	Biochemical efficacy: TG lowering	Established or unproven clinical outcomes	Major safety/tolerability issues	Main limitations in HTG/FCS
apoC‐III	Volanesorsen	ASO	Authorized in the European Union and some other regions as an adjunct to diet for adult patients with genetically confirmed FCS who remain at high risk of pancreatitis despite diet and conventional TG‐lowering therapy [415]; not approved by the US FDA [416]. Key trial: APPROACH (NCT02211209) [207].	In FCS, TG reductions of approximately 70%–80% have been reported [207].	Reduction in pancreatitis risk is mainly expected from TG lowering and supported by limited clinical evidence; ASCVD event reduction has not been established [415].	Injection‐site reactions are common; thrombocytopenia is the major treatment‐limiting toxicity and requires platelet monitoring [207, 415].	Safety concerns and monitoring burden may limit long‐term use.
apoC‐III	Olezarsen (TRYNGOLZA)	GalNAc‐conjugated ASO	Approved by the US FDA as an adjunct to diet to reduce TG levels in adults with FCS; subsequently approved to reduce TG levels and the risk of AP in adults with severe HTG, defined as fasting TG ≥500 mg/dL [417]. Key studies include BALANCE (NCT04568434) [33] and CORE/CORE2 (NCT05079919, NCT05552326) [418].	FCS: placebo‐adjusted TG reduction of approximately 43%. Severe HTG: placebo‐adjusted TG reductions of approximately 49–72% across pivotal trials [33, 417, 418].	In FCS, approval was primarily based on TG lowering, with reduction in AP events providing supportive evidence. In severe HTG, randomized controlled trial data and US FDA review support a reduction in AP events. ASCVD event reduction has not been established [33, 417, 418].	In FCS, common adverse reactions include injection‐site reactions, decreased platelet count, and arthralgia. In severe HTG, common adverse reactions include injection‐site reactions and liver enzyme elevations; hypersensitivity reactions and liver enzyme monitoring should be considered [417].	Indications and clinical benefits should be described separately for FCS and severe HTG. TG lowering is established, but ASCVD hard‐outcome benefit remains unproven.
apoC‐III	Plozasiran (REDEMPLO)	siRNA	Approved by the US FDA as an adjunct to diet to reduce TG levels in adults with FCS. Key study: PALISADE/Trial 1 (NCT05089084) [6, 419].	In FCS or persistent chylomicronemia, TG levels are substantially reduced; US FDA‐reported data showed a median TG reduction of approximately 59% versus placebo at Month 10, while trial‐level analyses reported larger within‐treatment reductions [6, 419].	Approval is based on TG lowering. A lower incidence of AP was observed in the pivotal trial, but reduction of AP risk is not included in the US FDA‐approved indication. ASCVD event reduction has not been established [6, 419].	Hyperglycemia, headache, nausea, and injection‐site reactions [419].	Current indication is limited to TG lowering in adults with FCS. Its role in multifactorial severe HTG, mixed dyslipidemia, and ASCVD risk reduction requires further study [419].
ANGPTL3	Evinacumab	Monoclonal antibody	Approved for HoFH; not approved for HTG or FCS. Evaluated in a Phase 2 study in severe HTG/chylomicronemia phenotypes (NCT03452228) [34, 420].	TG response is heterogeneous. TG lowering may occur in non‐FCS severe HTG phenotypes with residual LPL‐pathway activity, whereas efficacy is limited in biallelic LPL‐pathway–deficient FCS [34].	The severe HTG trial did not meet its prespecified primary endpoint; reductions in recurrent pancreatitis and ASCVD events have not been established [34].	Intravenous administration; efficacy depends on residual LPL‐pathway function. Cost and treatment burden associated with monoclonal antibody therapy should be considered [34, 420].	Not suitable as a general strategy for FCS caused by complete LPL‐pathway deficiency. It is not an approved treatment for HTG or FCS [34, 420].
ANGPTL4	MAR001	Monoclonal antibody	Completed Phase 1 and Phase 1b/2a studies; the Phase 2b TYDAL–TIMI 78 trial is recruiting. No Phase 2b outcome results are available as of June 2026 [421, 422].	In the Phase 1b/2a study, MAR001 produced dose‐dependent placebo‐adjusted mean TG reductions of approximately 41–53% at Week 12, with a maximal reported reduction of 52.7% [421].	Current evidence demonstrates short‐term improvement in lipid biomarkers only. No FCS‐specific efficacy data and no evidence yet for reductions in pancreatitis, hospitalization, or ASCVD events [421, 422].	Short‐term tolerability appears acceptable in early‐phase studies [421].	Still exploratory; long‐term safety, durability of effect, appropriate target population, and hard‐outcome benefits remain undefined.
LPL‐pathway restoration	Alipogene tiparvovec (Glybera)	AAV1‐mediated gene therapy	Previously authorized in the European Union for adult familial LPL deficiency with severe or multiple pancreatitis attacks despite dietary fat restriction; later withdrawn from the market after the marketing authorization was not renewed [423, 424].	Demonstrated disease‐modifying potential in monogenic LPL deficiency, with effects on TG levels and CM metabolism [425, 426].	Did not generate broadly generalizable long‐term clinical outcome evidence and is not a current mainstream treatment for HTG or FCS [423, 424, 426].	Very high cost, limited real‐world availability, and uncertainty regarding durability of benefit [424].	Primarily a translational proof‐of‐concept experience rather than a widely applicable current treatment strategy [423, 424, 426].

*Note*: This table distinguishes biochemical or lipid endpoints from clinical outcome benefits. Reductions in TG, apoC‐III, ANGPTL3, or RC are biochemical endpoints, whereas acute or recurrent pancreatitis, hospitalization, ASCVD events, mortality, and organ injury are clinical outcomes. Unless explicitly demonstrated in adequately designed clinical trials or included in regulatory labeling, marked TG lowering should not be interpreted as equivalent to a proven reduction in pancreatitis risk or ASCVD events. Regulatory status and approved indications are current as of June 2026 and may vary across jurisdictions. They should therefore be verified against the most recent regulatory documents and prescribing information. Regulatory terminology is reproduced as stated in the corresponding product labels. In particular, the US FDA indication for olezarsen uses the term severe hypertriglyceridemia for fasting TG levels ≥500 mg/dL, whereas the operational classification adopted in this review distinguishes severe HTG at 500–999 mg/dL from extreme HTG at ≥1000 mg/dL. Percentage reductions should not be compared directly across agents because study populations, baseline TG levels, doses, treatment durations, statistical methods, and reported efficacy measures differed among trials.

Abbreviations: AAV1, adeno‐associated virus serotype 1; ANGPTL3, angiopoietin‐like protein 3; ANGPTL4, angiopoietin‐like protein 4; AP, acute pancreatitis; apoC‐III, apolipoprotein C‐III; ASCVD, atherosclerotic cardiovascular disease; ASO, antisense oligonucleotide; CM, chylomicron; FCS, familial chylomicronemia syndrome; US FDA, US Food and Drug Administration; GalNAc, N‐acetylgalactosamine; HoFH, homozygous familial hypercholesterolemia; HTG, hypertriglyceridemia; LPL, lipoprotein lipase; RC, remnant cholesterol; siRNA, small interfering RNA; TG, triglyceride.

#### 6.5.1 ApoC‐III Inhibitors

ApoC‐III is an important negative regulator of TG homeostasis. It inhibits LPL‐mediated TRL lipolysis and reduces hepatic uptake of TRL remnant particles, thereby impairing TRL clearance [427]. Loss‐of‐function variants in APOC3 are associated with lower TG levels and lower ASCVD risk, providing genetic support for apoC‐III as a therapeutic target [138].

ApoC‐III inhibitors based on antisense oligonucleotide and small interfering RNA (siRNA) technologies have shown potent TG‐lowering effects in clinical studies, with some studies reporting TG reductions of 50–80%. In patients with FCS and some patients with extreme HTG, these agents can markedly reduce TG levels, and selected agents have also demonstrated reductions in AP events in high‐risk populations [6, 33]. Newer apoC‐III inhibitors have shown improved safety compared with earlier agents, particularly in reducing adverse events such as thrombocytopenia [428].

From a regulatory perspective, several apoC‐III‐targeted agents have received approval for specific high‐risk populations. Volanesorsen has been authorized in Europe, together with a low‐fat diet, for patients with genetically confirmed FCS who remain at high risk of pancreatitis despite diet and conventional TG‐lowering therapy [415]. Olezarsen was initially approved in the United States as an adjunct to diet to reduce TG levels in adults with FCS and was subsequently approved to reduce TG levels and the risk of AP in adults with severe HTG [429, 430]. Plozasiran has also been approved in the United States as an adjunct to diet to reduce TG levels in adults with FCS [431]. These regulatory indications primarily address severe chylomicronemia or extreme HTG in pancreatitis‐risk contexts and should not be directly extrapolated to mild‐to‐moderate HTG or ASCVD risk management without dedicated outcome evidence.

The clearest clinical role of apoC‐III inhibitors is in FCS, PC, and severe HTG associated with a high risk of pancreatitis, especially when the primary goals are to lower extremely high TG levels and prevent pancreatitis [432, 433]. Their effects on ASCVD events, long‐term benefits in multifactorial HTG, optimal timing of treatment, cost effectiveness, and long‐term safety require further investigation.

#### 6.5.2 ANGPTL3 Inhibitors

ANGPTL3 is an important regulator of lipid metabolism. It influences TG, HDL‐C, LDL‐C, and TRL metabolism   through its effects on LPL, endothelial lipase, and other pathways involved in lipoprotein metabolism [434, 435]. Monoclonal antibodies and RNA‐based therapies targeting ANGPTL3 have shown the ability to reduce TG, LDL‐C, and non‐HDL‐C levels [436, 437]. This makes ANGPTL3 an attractive potential target in the management of mixed dyslipidemia and residual cardiovascular risk [135, 438, 439].

Both apoC‐III and ANGPTL3 are involved in LPL regulation, but their mechanisms of action are not identical. ApoC‐III inhibits LPL and reduces hepatic uptake of remnant particles. In contrast, ANGPTL3 regulates both LPL and endothelial lipase and also affects lipoprotein remodeling [434, 440]. These pathways functionally overlap but are not fully redundant, suggesting that different HTG phenotypes may respond differently to targeted therapies.

The clinical role of ANGPTL3 inhibitors in the prevention of HTG‐AP and the reduction of HTG‐related ASCVD events remains incompletely defined. Evinacumab, an ANGPTL3 monoclonal antibody, has been approved for patients with homozygous familial hypercholesterolemia as an adjunct to other LDL‐C‐lowering therapies. It has not been approved as a routine treatment for HTG or HTG‐AP. Although existing studies support the ability of ANGPTL3 inhibition to improve some lipid parameters, evidence remains insufficient to establish reductions in the incidence of HTG‐AP, recurrence risk, and hard ASCVD endpoints [441, 442]. Therefore, when ANGPTL3‐targeted therapy is used in patients with HTG, improvement in lipid parameters should not be directly equated with clinical outcome benefit.

#### 6.5.3 Gene and Nucleic Acid‐Based Therapies

For LPL deficiency and related inherited forms of HTG, gene therapy and other nucleic acid‐based therapies remain important therapeutic directions [443]. Their therapeutic goals extend beyond lowering TG levels: gene replacement approaches aim to restore deficient LPL activity, whereas nucleic acid‐based therapies modulate genes involved in TRL metabolism to compensate for impaired lipid clearance without directly correcting the causal genetic variant. These approaches may reduce CM burden and pancreatitis risk and, potentially, modify the long‐term disease course [425].

Early translational studies explored strategies such as adeno‐associated viral vector‐mediated delivery of functional LPL genes. Other metabolic targets have also attracted interest because of their roles in TG synthesis and hepatic lipid export. These include diacylglycerol acyltransferase 1 (DGAT1) and diacylglycerol acyltransferase 2 (DGAT2). DGAT1 inhibition or DGAT2‐directed nucleic acid intervention has shown lipid‐lowering potential in some models of inherited HTG or metabolic disease [426, 444]. Apart from historical clinical experience with gene replacement, most of these approaches remain in the preclinical or early clinical stage. Their long‐term safety, durability of efficacy, tissue‐specific delivery, immunogenicity, cost effectiveness, and real‐world accessibility require further validation [445].

#### 6.5.4 Other Targets and Downstream Mechanism‐Based Interventions

Beyond direct targeting of TRL metabolism, downstream mechanisms of HTG‐related tissue injury have also emerged as potential therapeutic directions. Preclinical studies suggest that ferroptosis may contribute to inflammatory amplification and pancreatic injury in HTG‐AP. Ferroptosis inhibitors such as liproxstatin‐1 can attenuate lipid peroxidation, inflammation, and tissue injury in animal or cell models [165, 446, 447]. However, these interventions remain experimental, and there is currently insufficient human evidence to support their clinical use in the treatment of HTG‐AP.

Proprotein convertase subtilisin/kexin type 9 (PCSK9) inhibitors can mildly reduce TG levels in some studies and have demonstrated cardiovascular benefit in high‐risk ASCVD populations [448]. Their main mechanism is to enhance LDL receptor‐mediated clearance of LDL particles and other apoB‐containing lipoproteins, rather than to specifically correct severe HTG or chylomicronemia. Therefore, the role of PCSK9 inhibitors in HTG management is mainly limited to patients with a clear need for LDL‐C‐ or apoB‐related ASCVD risk management. They should not be regarded as primary TG‐lowering therapies for severe or extreme HTG or for the prevention of HTG‐AP [449, 450].

#### 6.5.5 Clinical Interpretation of Targeted Therapies

Novel targeted therapies, particularly apoC‐III‐ directed agents, have emerged as treatment options for selected patients with FCS, PC, and refractory severe or extreme HTG [451, 452]. When interpreting the clinical value of these therapies, three progressive levels should be considered: whether the drug can substantially lower TG levels or improve TRL metabolism; whether the drug has been approved for specific patient populations and in specific regulatory regions; and whether the drug has been proven to reduce clinical outcomes such as recurrent AP, ASCVD events, organ injury, or mortality. Inferring long‐term benefit solely from TG reduction is insufficient, especially because the dominant risks differ across HTG phenotypes.

### 6.6 Phenotype‐ and Risk‐Based Management Strategy

HTG management is stratified according to TG severity, lipoprotein phenotype, genetic background, and the dominant clinical risk. In patients with mild‐to‐moderate HTG and increased ASCVD risk, treatment relies on lifestyle intervention and statin therapy. Non‐HDL‐C, apoB, and overall ASCVD risk help determine whether high‐purity EPA, fibrates, or other therapies are added. In these patients, the goal is not simply to lower TG levels, but to reduce exposure to apoB‐containing particles and the long‐term risk of cardiovascular events.

In patients with severe or extreme HTG, particularly when chylomicronemia is present, or in those with a history of HTG‐AP, prevention of AP is a major therapeutic priority [2]. Management focuses on strict dietary control, alcohol abstinence, correction of diabetes and other secondary factors, and TG lowering with fibrates or omega‐3 fatty acids. ApoC‐III‐targeted therapy may be considered in refractory, recurrent, or inherited cases. Genetic evaluation is particularly relevant in patients with early‐onset, very severe, recurrent, or suspected monogenic forms of chylomicronemia to support phenotype identification, family management, and mechanism‐guided treatment [45].

For patients with both ASCVD risk and AP risk, an integrated strategy is needed to control both apoB‐containing particle burden and peak TG levels. Treatment goals are adjusted dynamically according to the current dominant risk. When TG levels are extremely high or AP risk is present, pancreatitis prevention takes priority. Once TG levels have been reduced to a safer range, clinical attention shifts back to long‐term ASCVD risk management, including control of non‐HDL‐C and apoB.

### 6.7 Challenges

Despite substantial progress in mechanistic research and drug development for HTG, clinical management still faces multiple challenges. In ASCVD risk management, many novel therapies can markedly reduce TG and TRL‐related markers. However, whether these therapies can further, consistently, and durably reduce ASCVD events on top of standard lipid‐lowering therapy remains insufficiently supported by high‐quality outcome evidence. The relative roles of TG, RC, non‐HDL‐C, and apoB in risk stratification and treatment target selection require further clarification [30, 453].

In the management of HTG‐AP, the evidence base remains limited, particularly regarding acute‐phase TG‐lowering interventions and strategies for preventing recurrence in patients with severe or extreme HTG, especially those with PC. Existing studies are mostly observational, single‐center retrospective analyses, or small cohort studies. For rapid TG‐lowering interventions such as TPE and intensive insulin therapy, the optimal indications, timing of initiation, stopping criteria, and true effects on hard clinical outcomes, including organ failure, AP recurrence, ICU length of stay, and mortality, have not been consistently established [454]. Acute‐phase treatment pathways for HTG‐AP still vary substantially across centers and urgently require standardization and validation in prospective studies.

Diagnostic and risk assessment criteria also need to be harmonized. Different studies and guidelines use partly inconsistent TG thresholds and terminology for pancreatitis risk, severe and extreme HTG, and chylomicronemia, as well as differing criteria for distinguishing FCS from MCS. This inconsistency limits cross‐study comparison and the uniformity of clinical decision‐making [455]. Future work should establish a stratification framework that integrates TG levels, apoB, non‐HDL‐C, RC, genetic background, secondary factors, and organ‐specific risk. Such a framework may more accurately distinguish pancreatitis‐predominant from ASCVD‐predominant phenotypes.

The real‐world implementation of emerging technologies and therapies remains challenging. AI‐assisted risk prediction models have shown potential [456, 457]. However, most models are still derived from single‐center, retrospective, and relatively small datasets, with insufficient external validation and limited interpretability [458, 459]. Novel targeted therapies and nucleic acid‐based therapies require further evaluation of long‐term safety, cost effectiveness, accessibility, patient selection criteria, and combination treatment strategies [414, 460]. Future HTG management should move beyond an approach driven by a single TG threshold and toward a precision treatment model based on etiology, lipoprotein composition, genetic background, and organ‐specific risk.

## 7 Conclusions and Future Perspectives

HTG is a highly heterogeneous metabolic disorder. Its clinical consequences are determined not only by the degree of TG elevation, but also by lipoprotein composition, apoB‐containing lipoprotein particles burden, genetic background, secondary factors, and the dominant clinical risk pathway [461]. HTG therefore represents a spectrum of disorders with different etiologic backgrounds, lipoprotein phenotypes, and clinical outcomes.

This heterogeneity has direct clinical implications. In HTG phenotypes dominated by ASCVD risk, especially persistent mild‐to‐moderate HTG, management should focus on reducing the overall atherogenic lipoprotein burden and long‐term ASCVD risk, rather than simply normalizing TG values. In phenotypes dominated by pancreatitis risk, particularly extreme HTG or HTG with chylomicronemia, the clinical priority should shift toward preventing abrupt TG elevation and persistent CM accumulation, thereby reducing the risk of HTG‐AP and its recurrence.

Increasing understanding of TRL metabolism, the LPL regulatory network, apoC‐III, ANGPTL3, RC, and lipotoxic inflammatory pathways has broadened the mechanistic framework and therapeutic landscape of HTG. Therapies targeting apoC‐III, ANGPTL3, and related pathways have emerged as approved or investigational treatment options for selected patients with FCS, PC, and refractory severe or extreme HTG. The efficacy of different targeted strategies may be influenced by the underlying genetic defect, residual LPL activity, and specific lipoprotein phenotype. Therefore, these approaches are unlikely to represent a uniform solution applicable to all patients with HTG.

A clear distinction remains necessary between biochemical efficacy and clinical benefit. Marked reductions in TG or TRL‐related biomarkers do not necessarily translate into reductions in HTG‐AP recurrence, ASCVD events, organ injury, or long‐term mortality. Future clinical trials need to enroll rigorously phenotyped patient populations and adopt clinical outcome‐centered study designs to validate the true benefits, safety, and cost effectiveness of emerging therapeutic strategies.

Several key questions remain unresolved, including phenotype‐specific thresholds for initiating intensive or targeted TG‐lowering therapy, the diagnostic boundary between FCS and MCS, optimal treatment sequencing and combination strategies, and the long‐term safety, accessibility, cost effectiveness, and clinical benefits of emerging therapies. Addressing these questions will require large‐scale, prospective, outcome‐oriented studies.

Future progress in the field of HTG will depend on the development of a multidimensional risk stratification system that moves beyond a single TG threshold. This system should integrate TG levels, apoB, non‐HDL‐C, RC, chylomicronemia status, genetic background, and secondary factors to assess pancreatitis risk and ASCVD risk separately. Genetic testing, multiomics analyses, longitudinal biomarkers, real‐world data, and AI‐assisted prediction models may help identify high‐risk phenotypes and predict treatment response. However, their clinical application still requires external validation, standardization, and prospective utility assessment.

Overall, HTG management is moving from “treating TG values” toward “identifying and treating risk phenotypes.” Integrating etiology, lipoprotein composition, genetic background, secondary factors, the dominant clinical risk, and treatment goals may support more precise risk stratification and individualized management. Such an approach may help prevent acute complications such as HTG‐AP, reduce residual ASCVD risk, and provide a more clinically practical pathway for improving both short‐term and long‐term outcomes in patients with HTG.

## Author Contributions

Shanshan Qi and Weiwei Chen: writing – original draft. Kunhao Chen and Song Yang: visualization. Sibo Wang: literature review. Enqiang Mao and Erzhen Chen: conceptualization and critical review. Ying Chen, Dan Xu, and Tongtian Ni: writing – review and editing. All authors have read and approved the final manuscript.

## Ethics Statement

The authors have nothing to report.

## Conflicts of Interest

The authors declare no conflicts of interest.

## Data Availability

The authors have nothing to report.
